# LSU network hubs integrate abiotic and biotic stress responses via interaction with the superoxide dismutase FSD2

**DOI:** 10.1093/jxb/erw498

**Published:** 2017-02-16

**Authors:** Antoni Garcia-Molina, Melina Altmann, Angela Alkofer, Petra M. Epple, Jeffery L. Dangl, Pascal Falter-Braun

**Affiliations:** 1 Technische Universität München (TUM), School for Life Sciences Weihenstephan (WZW), Plant Systems Biology, Emil-Ramann-Straße, 4, D-85354 Freising, Germany; 2 Howard Hughes Medical Institute and Department of Biology, University of North Carolina at Chapel Hill, Chapel Hill, NC 27599, USA; 3 BASF Plant Science LP, Research Triangle Park, NC 27709, USA; 4 Institute of Network Biology (INET), Helmholtz Zentrum München (HMGU), German Research Center for Environmental Health, 85764 Neuherberg, Germany; 5 Department of Microbe-Host Interactions, Ludwig-Maximilians-Universität München (LMU Munich), Planegg-Martinsried, Germany

**Keywords:** Abiotic stress, *Arabidopsis thaliana*, biotic stress, combinatorial stress, LOW SULPHUR UPREGULATED, LSU1

## Abstract

In natural environments, plants often experience different stresses simultaneously, and adverse abiotic conditions can weaken the plant immune system. Interactome mapping revealed that the LOW SULPHUR UPREGULATED (LSU) proteins are hubs in an Arabidopsis protein interaction network that are targeted by virulence effectors from evolutionarily diverse pathogens. Here we show that LSU proteins are up-regulated in several abiotic and biotic stress conditions, such as nutrient depletion or salt stress, by both transcriptional and post-translational mechanisms. Interference with *LSU* expression prevents chloroplastic reactive oxygen species (ROS) production and proper stomatal closure during sulphur stress. We demonstrate that LSU1 interacts with the chloroplastic superoxide dismutase FSD2 and stimulates its enzymatic activity *in vivo* and *in vitro*. *Pseudomonas syringae* virulence effectors interfere with this interaction and preclude re-localization of LSU1 to chloroplasts. We demonstrate that reduced LSU levels cause a moderately enhanced disease susceptibility in plants exposed to abiotic stresses such as nutrient deficiency, high salinity, or heavy metal toxicity, whereas LSU1 overexpression confers significant disease resistance in several of these conditions. Our data suggest that the network hub LSU1 plays an important role in co-ordinating plant immune responses across a spectrum of abiotic stress conditions.

## Highlight

The low molecular weight LOW SULPHUR UPREGULATED (LSU) network hubs mediate stomatal closure during several abiotic stress conditions by stimulating reactive oxygen species production; bacterial virulence effectors interfere with this function.

## Introduction

In nature and in farm fields, plants commonly encounter different forms of abiotic and biotic stress simultaneously. Due to global warming and the increasing need to farm on suboptimal soil, the incidence of combinatorial stress conditions is likely to increase. In molecular studies, stress responses are usually investigated in isolation. Although these approaches proved powerful for elucidating homeostatic response mechanisms for a variety of isolated stress conditions, it is becoming increasingly clear that the molecular responses to multiple simultaneous stressors differ from those to individual stressors. On a systems level, transcriptional profiling studies have demonstrated that the response to combinatorial stress qualitatively differs from the additive combination of single-stress responses (Atkinson *et al.*, 2013; Prasch and Sonnewald, 2013; Rasmussen *et al.*, 2013; Suzuki *et al.*, 2014). In particular, lack of nutrients, water shortage, or high salinity can weaken the plant immune system (Triky-Dotan *et al.*, 2005; Amtmann *et al.*, 2008; Al-Sadi *et al.*, 2010; You *et al.*, 2011; Kissoudis *et al.*, 2015).

Bacterial pathogens such as *Pseudomonas syringae* pv. *tomato* (*Pst*) can enter the apoplast through stomata, which mediate water and gas exchange and play a central role in many abiotic stress responses. During the first steps of infection, plants recognize conserved bacterial protein patterns such as flagellin and activate a primary immune response known as pattern-triggered immunity (PTI), which includes increased reactive oxygen species (ROS) production, callus deposition, and other defence mechanisms (Jones and Dangl, 2006). Adapted pathogens can overcome initial immune responses by delivering virulence effector proteins into host cells that reach different subcellular compartments and interfere with PTI. Plants conversely possess intracellular receptors to detect the presence of virulence effectors by direct or indirect recognition, and trigger enhanced immune responses resulting in effector-triggered immunity (ETI). Beyond the idealized zig-zag model of the plant immune system (Jones and Dangl, 2006), it is clear that the perpetual arms race between pathogens and their hosts has resulted in a highly complex quantitative interplay of defence and counter-defence mechanisms.

To identify host interaction partners of virulence effectors and thus potentially novel components of the plant immune system, we recently conducted large-scale protein interaction mapping experiments between Arabidopsis proteins and virulence effectors from three evolutionarily distant pathogens. We discovered that effectors from *Pst*, the oomycete *Hyaloperonospora arabidopsidis*, and the fungus *Golovinomyces orontii* converge onto common host proteins, several of which are hubs in the plant protein network (Arabidopsis Interactome Mapping Consortium, 2011; Mukhtar *et al.*, 2011; Wessling *et al.*, 2014). Hubs in binary interaction networks were previously shown to mediate and integrate diverse cellular processes (Han *et al.*, 2004; Yu *et al.*, 2008). Moreover, we observed a correlation between the extent of effector convergence onto host targets and the manifestation of immune phenotypes in the corresponding genetic nulls (Mukhtar *et al.*, 2011; Wessling *et al.*, 2014). Incorporating population genetic data, we further discovered that protein products of Arabidopsis genes that experience the strongest balancing and positive selection preferentially interact with effector-targeted proteins (Wessling *et al.*, 2014). Thus, despite the fact that no immune function had previously been described for most of the new effector targets, our collective data suggest that these are important in host–pathogen interactions.

Among the highly targeted host proteins are Arabidopsis LOW SULPHUR (S) UPREGULATED (LSU) proteins. *Arabidopsis thaliana* has four members of the LSU protein family (LSU1–LSU4), which can be found in all higher land plants. *LSU* genes were named for their strong transcriptional induction in response to S deficiency (–S) (Maruyama-Nakashita *et al.*, 2003), and a general function for the *Nicotiana benthamiana* LSU orthologue UP9C during –S stress was demonstrated (Lewandowska *et al.*, 2010). However, the molecular and precise physiological functions of LSU proteins remain to be clarified. The observation that LSU family members are intensely targeted by evolutionarily diverse pathogens (Mukhtar *et al.*, 2011; Wessling *et al.*, 2014) suggests a hitherto unknown function in plant immunity.

Here we report that *LSU1* and *LSU2* levels are increased by transcriptional and post-translational mechanisms in a variety of abiotic stress conditions. In response to –S or salt stress, *LSU* gene expression is required for ROS production in guard cell chloroplasts and subsequent stomatal closure. Using genetic and biochemical approaches we demonstrate that LSU1, which is expressed in guard cells, physically interacts with the iron (Fe)-dependent superoxide dismutase (SOD) FSD2 and can activate its enzymatic activity. Virulence effectors from *P. syringae* interfere with the function and subcellular localization of LSU1. Correspondingly, in conditions of abiotic stress, we observe a moderate enhanced disease susceptibility (EDS) phenotype in seedlings with reduced *LSU* levels and a corresponding enhanced disease resistance (EDR) phenotype in LSU1 overexpressors. Together we uncover a physiological function for LSU proteins during combinatorial biotic and abiotic stress and propose a working mechanism of action for LSU1.

## Materials and methods

### Plant cultivation and manipulation

*Arabidopsis thaliana* Col-0 was cultivated on half-strength Murashige and Skoog (1/2 MS) medium or with S compounds substituted by equivalent chloride salts and supplemented with 0.5% (w/v) MES and Plant Preservative Mixture at 0.04% (v/v; Plant Cell Technology) under long-day conditions (16 h light–21 °C/8 h dark–16 °C). For stress treatments 7-day-old wild-type (WT) seedlings grown on 1/2 MS plates were transferred to liquid 1/2 MS medium without S or Fe or with 10 µM Cu, 2 mM DTT, 150 mM NaCl, or pH 8 for 1 d. For ROS detection and *P. syringae* assays, seedlings were grown on standard medium, without S, or in the presence of 25 µM Cu or 50 mM NaCl. Stable transgenic lines were generated with the *Agrobacterium tumefaciens* GV3101 (pMP90) strain by floral dipping (Koncz and Schell, 1986; Clough and Bent, 1998). The *fsd2*-2 mutant corresponds to the SALK line SALK_080457C (ID NASC: N663088) (Myouga *et al.*, 2008).

### Gene expression analysis

RNA was prepared with the NucleoSpin RNA kit (Macherey-Nagel) following the manufacturer’s instructions. RNA quantity and quality was evaluated by spectrometry and in agarose gels prior to reverse transcription to cDNA using M-MLV Reverse Transcriptase, RNase H Minus, Point Mutant (Promega). Quantitative PCRs (qPCRs) were conducted using the SsoAdvanced Universal SYBR Green Supermix (Bio-Rad) in a CFX96 Touch™ Real-Time PCR Detection System (Bio-Rad) using an initial cycle at 95 °C for 3 min and 40 cycles consisting of 95 °C for 10 s, 58 °C for 20 s, and 72 °C for 20 s. To assess expression changes of *LSU* genes, the measured levels were first normalized to *ACTIN2* (*ACT2*) and *ELONGATION FACTOR1* (*EF1*) levels, except for salt and pH 8 treatments where these controls showed strong regulation and where *ACT8* and *UBIQUITIN10* (*UBQ10*) were used instead. The normalized expression levels are represented as fold change in expression relative to control conditions.

### Protein analysis and biochemical fractionation

Total protein extracts were prepared in 100 mM NaCl; 50 mM Tris–HCl pH 7.5; 0.5% (v/v) Triton X-100; 1 mM DTT; 1× Complete Protease Inhibitor Cocktail Tablet (Roche), and spectrophotometrically quantified using ROTIQUANT (Carl Roth). A 10–20 µg aliquot of protein extract was loaded onto an SDS-polyacrylamide gel, blotted on nitrocellulose membranes, probed with antibodies listed in Supplementary Table S3 at *JXB* online, and developed with the SuperSignal West Femto Chemiluminescent Substrate (Thermo Scientific) in a Luminescent Image Analyzer LAS4000 System (Fujifilm). Biochemical fractionation was conducted as previously described (Garcia-Molina *et al.*, 2014) from 7-day-old *CaMV35S:HA-LSU1/2* seedlings in a Sorvall™ MTX 150 Micro-Ultracentrifuge (Thermo Scientific) with a S55A2 rotor. Fractions were assayed by western blots with antibodies anti-H3, anti-UGPase, anti-V-ATPase, and anti-haemagglutinin (HA) (Supplementary Table S3).

### Subcellular localization experiments

*CaMV35S:GFP-LSU1/2* and *CaMV35S:GFP* (Cutler *et al.*, 2000) transgenic seedlings were examined in a FLUOVIEW FV1000 confocal laser microscope (Olympus) using specific green fluorescent protein (GFP) and chlorophyll filters.

### Generation of constructs and amiRNA lines

Full-length ORFs were available as GATEWAY™ entry clones (Arabidopsis Interactome Mapping Consortium, 2011). To generate constructs for GFP and HA fusion proteins, entry clones were transferred into *pMDC43* or *pALLIGATOR2*, respectively, using GATEWAY recombination reactions (Life Technologies) (Curtis and Grossniklaus, 2003; Bensmihen *et al.*, 2004). For bimolecular fluorescence complementation (BiFC) assays, *pYFN43* and *pYFC43* were used (Belda-Palazón *et al.*, 2012). Translational fusions to glutathione *S*-transferase (GST) and maltose-binding protein (MBP) were generated with *pGEX6* and *pMAL-DEST* plasmids (Braun *et al.*, 2002), respectively. Artificial miRNAs (amiRNAs) targeting *LSU1–LSU4* were designed as in Schwab *et al.* (2006) with the oligonucleotides listed in Supplementary Table S2, GATEWAY-recombined into *pDONR207* entry vector (Life Technologies), and subsequently into *pALLIGATOR3* (Bernaudat *et al.*, 2011).

### Stomatal aperture and water loss determination

To determine stomatal aperture, 12-day-old Arabidopsis seedlings grown on S-sufficient or -deficient media were transferred to the indicated media supplemented with 10 µM abscisic acid (ABA; Sigma-Aldrich), or the corresponding mock treatment, for 3 h. First leaflets were abaxially observed by light microscopy, and the ratio between the width and length of ostiols (R_wl_) was measured. Water loss of 12-day-old seedlings was inferred from the change in fresh weight over 60 min after removing seedlings from plates. For each biological replicate, at least 30 stomata were analysed.

### ROS production in guard cells

ROS production was traced as previously described (Miao *et al.*, 2006; Zou *et al.*, 2015) with modifications. Briefly, detached first leaflets of 14-day-old seedlings were incubated for 10 min on 50 µM H_2_DCFDA (Life Technologies), washed twice with water, and observed either under laser confocal (Olympus FV1000) or BX61 epifluorescence microscopy (Olympus) with a GFP band-pass filter. 3,3’-Diaminobenzidine (DAB) staining was used to detect H_2_O_2_ specifically. To this end, 14-day-old seedlings were incubated with 1 ml of DAB solution [1 mg ml^–1^ (w/v) DAB; 0.05% (v/v) Tween-20; 10 mM Na_2_HPO_4_] and vacuum-infiltrated for 5 min in the dark. Samples were left at room temperature for 5 h protected from the light, and de-stained with two washes with ethanol:acetic acid:glycerol (3:1:1) solution prior to microscopy.

### Production of recombinant proteins

*Escherichia coli* Rosetta™ (Novagen) transformants were grown to log phase (OD_600_ 0.4–0.8) in LB medium supplemented with antibiotics and with 0.2% glucose for MBP constructs; protein expression was induced with 1 mM isopropyl-β-d-thiogalactopyranoside (IPTG) for 4 h at 28 °C. Pelleted cells were resuspended in 1× phospahte-buffered saline (PBS) supplemented with 1× Complete Protease Inhibitor Cocktail Tablet (Roche), sonicated, and lysates were rotated with either Amylose Resin (New England Biolabs) or Protino Glutathione Agarose 4B (Macherey-Nagel) for 2 h at 4 °C. The matrix was washed twice with 3 vols of PBS or MBP wash buffer (200 mM NaCl; 20 mM Tris–HCl pH 7.4; 1 mM EDTA; 1 mM DTT), respectively, and eluted with 2 vols of 10 mM Tris–HCl pH 8, 10 mM glutathione, or MBP wash buffer supplemented with 10 mM maltose.

### Protein–protein interaction assays

Yeast two-hybrid (Y2H) experiments were conducted with the indicated ORFs cloned into *pDEST-AD* or *pDEST-DB* as described (Dreze *et al.*, 2010). BiFC assays were carried out by agro-injection of *pYFN43-LSU1/2* and *pYFC43-FSD2*, *pYFC43-PTEN1*, or *pYFC43-LSU1/2* in *N. benthamiana* epidermal cells at OD_600_ 0.1 and observed after 1–2 d under epifluorescence microscopy (Olympus BX61) with a yellow fluorescent protein (YFP) band-pass filter. For *in vitro* pull-down assays, Protino Glutathione Agarose 4B (Macherey-Nagel) alone or coated with GST–FSD2 was incubated with equimolar combinations of purified MBP–LSU1 and/or MBP–NIMIN1, MBP–AvrXccC, MBP–AvrB2, or MBP–HopR1 in a final volume of 100 µl of PBS and incubated with gentle rotation for 3 h at 4 °C. Beads were washed four times with 3 vols of PBS each and eluted with 10 mM Tris–HCl pH 8, 10 mM glutathione. MBP–LSU1 pull-downs were analysed by western blot using anti-MBP antibodies (Supplementary Table S3).

### Determination of superoxide dismutase enzymatic activity

SOD activity was determined as described by Kuo *et al.* (2013) with modifications. Briefly, samples were incubated with 1 ml of reaction buffer [5 mM Nitro Blue Tetrazolium (NBT); 50 mM PBS pH 7.8; 10 mM methionine; 0.5 mM EDTA; and 20 µM riboflavin] for 30 min in the dark, and then illuminated for 10–15 min. SOD activity was monitored by measuring inhibition of NBT–diformazan formation at *A*_560_. *Nicotiana benthamiana* samples were prepared in extraction buffer [50 mM PBS pH 7.8; 0.05% (v/v) NP-40; 2% (w/v) polyvinylpolypyrrolidone (PVPP); 1 mM EDTA]. For *in vitro* assays, purified recombinant GST–FSD2 (3 µg) or GST–FSD1 (1.5 µg) were incubated with the indicated recombinant proteins in 50 µl of PBS at room temperature for 30 min prior to enzymatic activity determination.

### *Pseudomonas syringae* infection assays

Infection assays were conducted as previously described in Ishiga *et al.* (2011). *Pseudomonas syringae* pv. *tomato* DC3000, COR^–^, or *hrcC* was cultured overnight at 28 °C in NYGA medium [0.5% (w/v) bactopeptone, 0.3% (w/v) yeast extract; 2% (v/v) glycerol] supplemented with antibiotics. Infection assays were performed on 12-day-old seedlings grown *in vitro* by flooding with bacterial suspension at OD_600_ 0.01 (5 × 10^6^ CFU ml^–1^) for 3 min. *Pseudomonas syringae* density was determined after 3 d. For this, shoots were harvested, disinfected with 5% (w/w) H_2_O_2_, and rinsed twice with water. Samples were ground and spotted in serial dilutions on selective medium (LB supplemented with 50 mg l^–1^ rifampicin and 30 mg l^–1^ kanamycin for all strains and also 30 mg l^–1^ spectinomycin for COR^–^) to obtain colony counts after 1 d of incubation at 28 °C. To ensure equal infection rates, WT and amiR-LSU lines were cultivated together.

### Miscellaneous methods

Student’s *t*-test was used to determine significant differences from control lines or treatments as indicated. Fluorescence and band intensities were quantified with the image-processing package Fiji. Sequence analysis and multiple alignments were performed with the CLC Sequence Viewer 7 (Qiagen) software. Protein distance measure is indicated according to Jukes and Cantor (1969).

## Results

### Transcriptional and post-translational regulation of LSU levels in abiotic stress

**Fig. 1.**
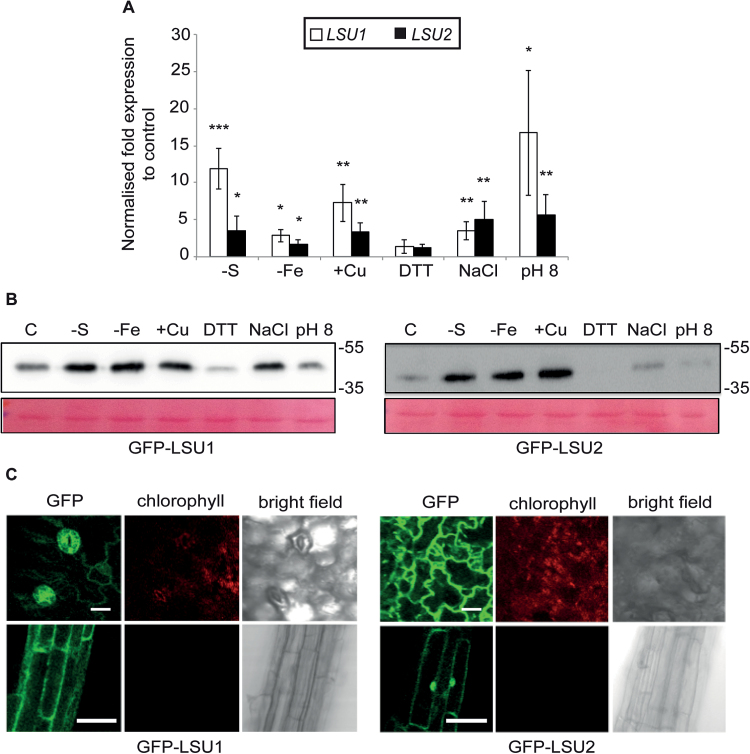
Characterization of LSU1 and LSU2. (A and B) LSU1 and LSU2 expression is induced under certain abiotic stress conditions. (A) Expression of *LSU1* and *LSU2* transcripts in different abiotic stress conditions was determined by qPCR and expressed as the ratio between experimental and control treatments. Error bars correspond to the SD of three biologically independent measurements, and significant differences were assessed by the Student’s *t*-test relative to control treatments (*P<0.05; **P<0.01; ***P<0.001). (B) α-GFP western blots on total lysates of GFP–LSU1- and GFP–LSU2-expressing seedlings grown as indicated. Ponceau staining shows the large RuBisCO subunit and indicates equal loading; molecular weight marker sizes are indicated in kDa. (C) Representative confocal images of cotyledons and roots of *GFP–LSU1* and *GFP–LSU2* seedlings grown on –S media showing the GFP signal, chlorophyll autofluorescence, and bright field are provided (scale bar=50 µm).

Hubs in binary interaction networks were shown to co-ordinate different cellular processes (Han *et al.*, 2004; Yu *et al.*, 2008). Therefore, we wondered whether *LSU* genes might also function in abiotic stress conditions other than –S conditions (Nikiforova *et al.*, 2003; Arabidopsis Interactome Mapping Consortium, 2011; Hawkesford *et al.*, 2012. We investigated transcript and protein abundance in several abiotic stress conditions selecting LSU1 and LSU2 (LSU1/2) as representative members of the family (Supplementary Fig. S1A–C). *LSU1*/*2* transcript levels were determined by qPCR in 7-day-old WT seedlings transferred to liquid media representing different abiotic stress conditions. In addition to the induction by –S, *LSU* transcript levels increased between 2- and 15-fold in WT seedlings exposed to increased salinity, iron (Fe) deficiency, copper (Cu) excess, or basic pH; no change was observed in response to DTT (Fig. 1A). To investigate potential post-translational regulation in the absence of transcriptional changes, we used transgenic lines expressing LSU1/2 fusion proteins from ectopic promoters. *CaMV35S:GFP-LSU1/2* transgenic seedlings accumulated more GFP–LSU1 upon –S, Fe deprivation, Cu excess, and salt excess in comparison with control treatments, whereas increased GFP–LSU2 amounts were found on –S, Fe deficiency, and high Cu, but not salt excess. For the rest of the treatments, no differences or even reduced levels were observed (Fig. 1B). The LSU1 and LSU2 responsiveness towards several abiotic stress conditions suggests that LSU proteins could mediate plant responses to a variety of abiotic stress conditions.

### LSU1 and LSU2 display differential subcellular and tissue localizations

Despite the high similarity of LSU proteins (Supplementary Fig. S1A–C), the interaction partners of LSU1/2 proteins previously identified in the Arabidopsis interactome only partially overlap (Arabidopsis Interactome Mapping Consortium, 2011). We wondered whether the localizations of LSU proteins further support a functional specialization and interrogated the tissue and subcellular localization of LSU1/2 *in planta*. A biochemical fractionation following heterologous expression in *N. benthamiana* showed that LSU1/2 can be found in nuclear, cytosolic, and microsmal fractions (Supplementary Fig S2A, B). We therefore investigated the tissue and subcellular localization of GFP–LSU1/2 proteins in Arabidopsis. Microscopically, *GFP–LSU1* lines display a diffuse fluorescent signal in the root cytoplasm/membrane (Fig. 1C, lower panel, left). In leaflets of 5-day-old seedlings, GFP–LSU1 was prominently detected in guard cells, suggesting a function in stomatal regulation (Fig. 1C, upper panel, left). GFP–LSU2 exhibited a diffuse but ubiquitous cytosolic signal in leaves and roots, and also illuminated nuclei in root cells (Fig. 1C, right). Soluble GFP alone showed an overall similar pattern, which is expected given the small size of LSU proteins and GFP. Nonetheless, clear differences can be seen especially in the strong stomatal localization of GFP–LSU1 compared with pavement cells and more prominent nuclear localization of LSU2 in root cells (Fig. 1C; Supplementary Fig. S2C). The results support the conclusion that LSU proteins can localize to multiple cell compartments and suggest at least partially specialized and tissue-specific roles for LSU1 and LSU2. Importantly, the data point towards a likely function of LSU1 in guard cells. This conclusion is supported by markedly reduced *LSU1* transcript levels in cotyledons of the stomataless mutants *spch-3* and *mute-3* (de Marcos *et al.*, 2015).

### Abrogation of *LSU* expression constrains stomatal movements upon sulphur deficiency

No T-DNA mutants were available for *LSU1* and *LSU3*, and the paired duplications of the *LSU1/3* and *LSU2/4* paralogues further challenge generation of higher order mutants. Hence, to characterize the *LSU* genes phenotypically and avoid problems due to potential redundancy, three different amiRNAs targeting all *LSU* family members at different positions were designed and used to generate three amiR-LSU lines (amiR-LSUa–c; Supplementary Fig. S3A). Transcript levels of the four *LSU* genes were determined by qPCR in seedlings grown on –S. Compared with WT lines, *LSU1–LSU3* transcripts were reduced by >80% and *LSU4* levels by 50% in the amiR-LSUa–c (Supplementary Fig. S3B). Based on these results, the amiR-LSU lines were considered knock-down lines and used for subsequent phenotypic studies.

**Fig. 2.**
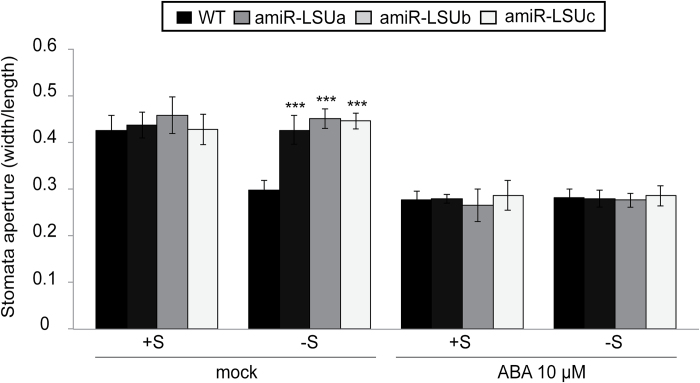
Abrogation of LSU constrains stomatal closure during abiotic stress conditions. Twelve-day-old WT and amiR-LSUa–c seedlings grown in +S or –S conditions were transferred to the same media supplemented with ABA or mock treated for 3 h. Stomatal aperture was quantified as the ratio of ostiol width to length (R_wl_). Error bars correspond to the SD of ≥3 biologically independent measurements (*n* ≥30 stomata), and significant differences were assessed by the Student’s *t*-test relative to the WT in the same conditions (***P<0.001).

The amiR-LSU lines did not exhibit obvious phenotypes on soil or *in vitro* culture in comparison with the WT (data not shown). Since GFP–LSU1 localized to guard cells and stomatal closure is a physiological response to –S (Terry, 1976; Karmoker *et al.*, 1991), guard cell movements in the amiR-LSU lines were characterized. The apertures of abaxial stomata of first leaflets were estimated as the ratio between the width and length of the ostiols (R_wl_). Stomata were mostly open (R_wl_ ~0.45) in both WT and amiR-LSUa–c lines grown on S-sufficient (+S) media. S deficiency, however, led to partial stomatal closure in WT lines indicated by an ~30% decrease in R_wl_. In contrast, stomata remained open in amiR-LSU lines in –S media (Fig. 2A; Supplementary Fig. S4A), indicating that stomatal closure during –S conditions is an LSU-dependent process. In agreement with these observations, the amiR-LSUa–c lines exhibited a more severe water loss on –S media compared with the WT (Supplementary Fig. S4B; Supplementary Table S1). Notably, stomatal closure in response to exogenous ABA was not impaired in either condition, indicating that ABA-dependent stomatal closure is not affected in the amiR-LSU lines (Fig. 2). Thus, LSU proteins, probably the prominently guard cell-localized LSU1, participate in the reg4ulation of stomatal closure in response to –S.

### *LSU* down-regulation compromises ROS production in guard cells upon certain abiotic stress conditions

**Fig. 3.**
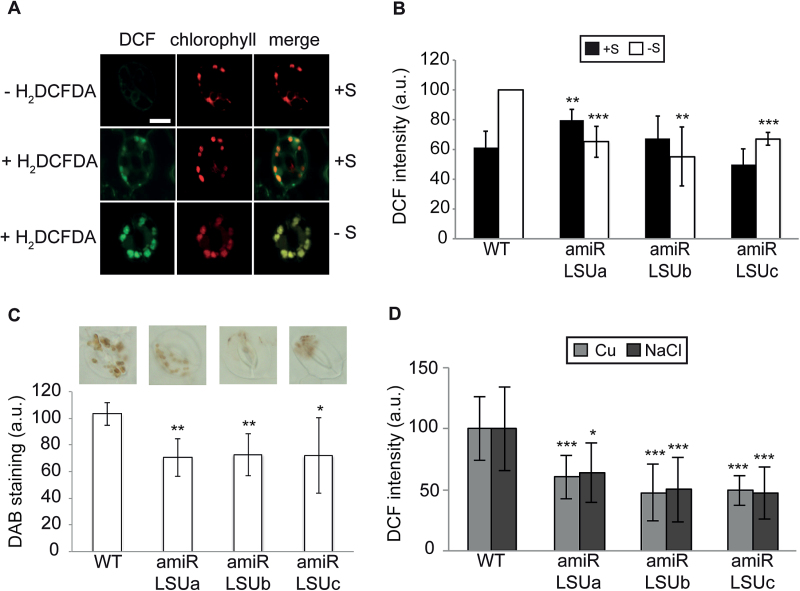
Abrogation of LSU impairs ROS production during abiotic stress conditions. (A) Reactive oxygen species (ROS) in chloroplast guard cells increase during sulphur starvation. Representative confocal pictures of the abaxial guard cell stomata of WT seedlings grown under +S or –S conditions showing DCF and chlorophyll fluorescence and a merged signal (scale bar=10 µm). (B) Quantification of relative DCF fluorescence intensity of stomata (*n* ≥30) of WT and amiR-LSUa–c seedlings grown in +S and –S conditions. (C) Hydrogen peroxide production in guard cells of amiR-LSU lines is compromised during sulphur starvation. Representative pictures of 3,3’-diaminobenzidine (DAB) staining in guard cells of seedlings grown on –S and quantification of signal intensity per guard cell pair. (D) ROS production in chloroplast guard cells is compromised during copper and salt treatments. Quantification of DCF signal intensity in guard cells of seedlings grown on 25 µM CuCl_2_ and 50 mM NaCl, respectively. In all cases, error bars correspond to the SD of ≥3 biologically independent measurements, and significant differences were assessed by the Student’s *t*-test relative to the WT in the same conditions (*P<0.05; **P<0.01; ***P<0.001).

Guard cell movements are controlled by a complex interplay of hormones and secondary signals (Daszkowska-Golec and Szarejko, 2013; Arnaud and Hwang, 2014), which culminate in an increase of H_2_O_2_ that triggers ion channel opening and a change in guard cell turgor (Pei *et al.*, 2000; Zhang *et al.*, 2001; Desikan *et al.*, 2004; Wang *et al.*, 2012). To address whether ROS production during –S stress is impaired in guard cells, we pharmacologically monitored ROS production using the ROS-mediated conversion of 2’,7’-dichlorodihydro-fluorescein diacetate (H_2_DCFDA) into fluorescent 2’,7’-dichlorofluorescein (DCF) (Brandt and Keston, 1965). In +S conditions, DCF fluorescence in guard cells exhibited a diffuse pattern partly co-localizing with chloroplast autofluorescence (Fig. 3A). In contrast, in –S conditions, DCF fluorescence intensity nearly doubled (~40%) compared with +S and exclusively localized to speckles overlapping the chlorophyll autofluorescence (Fig. 3A, B). In the amiR-LSU lines, the –S-induced stimulation of ROS production was either dramatically reduced (amiR-LSUc) or completely abrogated (amiR-LSUa, b; Fig. 3B; Supplementary Fig. S4C). Among the various ROS species, H_2_O_2_ is of particular interest due to its varied roles as a defence and signalling molecule. To test if the observed differences in guard cell ROS production could be attributed to an H_2_O_2_ accumulation, DAB staining on S-deprived seedlings was conducted and the signal intensity per guard cell pair quantified. As depicted in Fig. 3C, the amiR-LSUa–c lines showed a reduced DAB staining pattern (~30%) compared with the WT. In addition, we tested whether ROS production in the amiR-LSU lines is affected in conditions other than –S. Consistent with the induction of *LSU* transcripts in response to high salt or Cu, the amiR-LSU lines exhibited 40–50% lower DCF signal intensity compared with the WT (Fig. 3D). These data demonstrate that LSU proteins stimulate ROS production, among them H_2_O_2_, in guard cell chloroplasts in response to different abiotic stress conditions.

### LSU1 physically interacts with and activates the iron superoxide dismutase FSD2

**Fig. 4.**
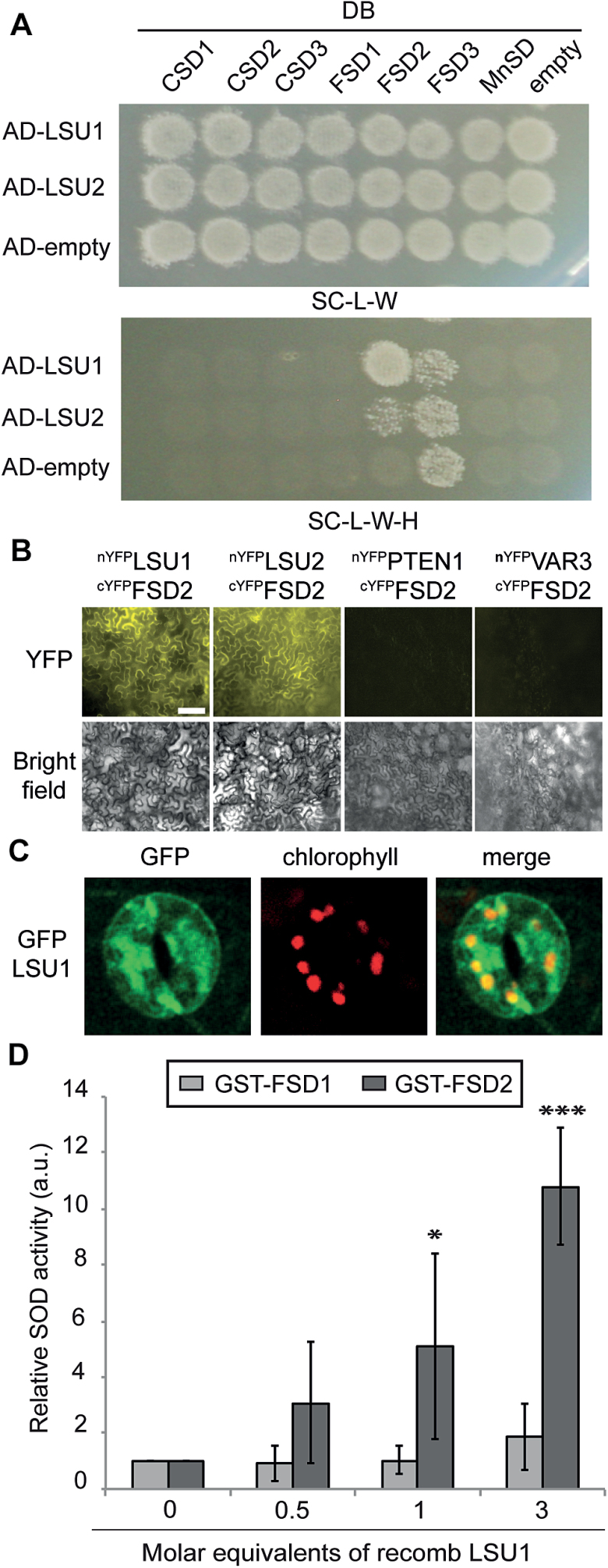
LSU1 and LSU2 physically and functionally interact with FSD2. (A) Specific interaction of AD–LSU1 and AD–LSU2 with DB–FSD2 by Y2H. The top panel shows diploid yeasts and successful mating; the bottom panel shows growth on selective media indicating interactions of AD–LSU1/2 with DB–FSD2 and autoactivation of DB–FSD3. (B) Bimolecular fluorescence complementation (BiFC). *Nicotiana benthamiana* epidermal leaves transiently co-expressing cYFP–FSD2 and nYFP–LSU1/2 restore YFP fluorescence, whereas co-infiltration of cYFP–FSD2 and nYFP fusions with the cytosolic Arabidopsis PHOSPHATASE AND TENSIN HOMOLOG DELETED ON CHROMOSOME TEN 1 (PTEN1, AT5G39400) and with the chloroplast-localized nYFP–VARIEGATED 3 (VAR3, AT5G17790) do not (scale bar=100 µm). (C) LSU1 partly co-localizes with guard cell chloroplasts. Representative confocal images of guard cells in first leaflets of –S-grown *GFP–LSU1* seedlings. Shown are GFP signal (left), chlorophyll autofluorescence (middle), and merged channels (right) (scale bar=25 µm). (D) Relative *in vitro* SOD activity of recombinant GST–FSD1 and GST–FSD2, respectively, in the presence of increasing concentrations of MBP–LSU1. Error bars correspond to the SD of three independent experiments, and significant differences from controls were assessed using Student’s *t*-test (*P<0.05; ***P<0.001).

H_2_O_2_ mainly occurs in cells as a result of the enzymatic conversion of the superoxide radical (O_2_^–^) by SODs. Arabidopsis possesses three different SOD isoforms classified according to their respective metal cofactors: manganese (MnSOD), Cu/zinc (CSD1/2), and Fe (FSD1–FSD3) (Kliebenstein *et al.*, 1998). Of these, FSD2, FSD3, and CSD2 localize to plastids and may therefore mediate the H_2_O_2_ production during –S stress, whereas the others function in different cell compartments. We previously identified FSD2 as an interaction partner of LSU1 and LSU2 (Arabidopsis Interactome Mapping Consortium, 2011) and aimed to verify this interaction; to ascertain its specificity, we also included all other Arabidopsis SOD proteins in this verification experiment. Y2H experiments using GAL4 activation domain (AD)–LSU1/2 and all Arabidopsis SODs fused to the GAL4 DNA-binding domain (DB) confirmed the specific interaction of LSU1/2 with the chloroplast-localized FSD2 (Fig. 4A). Due to self-activation of FSD3, a possible association with LSU1/2 could not be tested (Fig. 4A). BiFC in *N. benthamiana* demonstrates that LSU1 and LSU2 can interact with FSD2 *in planta* (Fig. 4B). Additional support for the interactions was obtained in biochemical pull-down experiments (see below). Moreover, upon closer inspection of confocal images, we found that in –S conditions GFP–LSU1 partly co-localized with chlorophyll autofluorescence in guard cells, suggesting plastidial localization of LSU1 in these conditions [Pearson correlation coefficient (PCC) 0.52; Fig. 4C]. Thus, LSU1 can physically interact with FSD2 *in vitro* and *in vivo*.

**Fig. 5.**
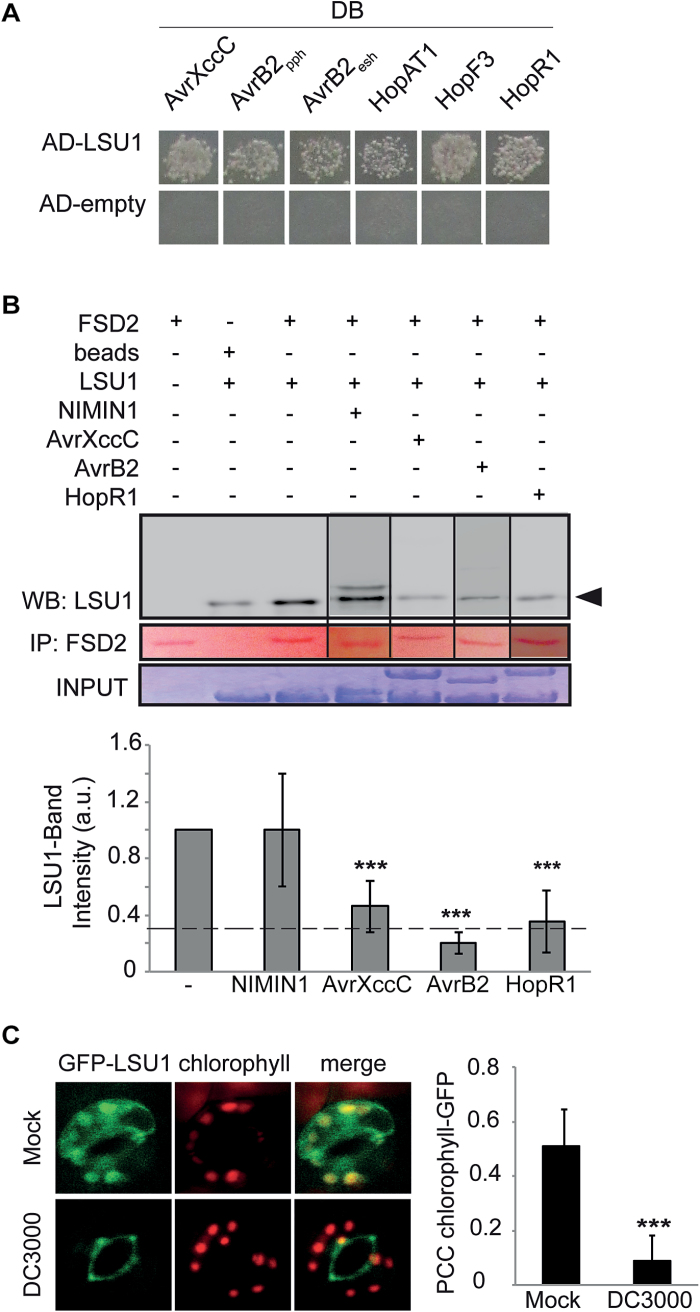
*Pseudomonas* virulence effectors interact with LSU1 and interfere with its functions. (A) Interactions in the Y2H system between AD–LSU1 and DB fusions with bacterial virulence effectors: *Xcc* AvrXccC (G9BA82_XANCA), *Psp* AvrB2 (PSPPH_A0120, AvrB2_pph_ and A2BCU4_PSESH, AvrB2_esh_), *Pst* HopAT1 (PSPTO_5618), HopF3 (PSPTO_0502), and HopR1 (PSPTO_0883, C-terminal region). (B) Representative α-MBP western blot analysis of pull-down experiments of recombinant MBP–LSU1 by GST–FSD2 in the presence of the negative control protein MBP–NIMIN1 or the MBP-tagged virulence effectors AvrXccC, AvrB2 (AvrB2_pph_), and HopR1 (C-term). Ponceau staining shows precipitated GST–FSD2. Coomassie brilliant blue (CBB) shows input material. Quantification of precipitated MBP–LSU1 relative to no competitor summarizing ≥3 independent experiments is shown in the lower panel. For all quantifications, the no-competitor positive control was loaded on the same gel. The dashed line indicates background MBP–LSU1 in the beads-only control. (C) Representative confocal images of *GFP–LSU1* seedlings grown on –S after *Pst* or mock infection; shown are GFP fluorescence, chlorophyll autofluorescence, and merged channels. The bar graph shows the PCC of the GFP and chlorophyll fluorescent patterns in *Pst*- and mock-infected leaves (*n* >10). In (B, lower panel) and (C) the significance of differences was assessed using the Student’s *t*-test (*P<0.05; ***P<0.001).

Given that reduced LSU levels lead to decreased ROS production in guard cells under abiotic stress conditions, we wondered if LSU proteins could directly stimulate FSD2 enzyme activity. *In vivo* activity measurements using *N. benthamiana* leaves transiently expressing GFP–LSU1 and GFP–FSD2 together or alone showed only incremental stimulation of FSD2 activity by LSU1 (Supplementary Fig. S5A, B). To eliminate confounding effects by endogenous proteins, we purified recombinant proteins and investigated the effect of LSU1 and LSU2 on FSD2 activity *in vitro*, using the non-interacting FSD1 to assess specificity (Fig. 5A). The functionality of GST-tagged FSD1 and FSD2 purified from *Escherichia coli* was confirmed by plotting SOD activity against the enzyme concentration, which yielded classic saturation curves (Supplementary Fig. S5C). Non-saturating enzyme concentrations were then incubated with increasing amounts of MBP–LSU1, and SOD activity was determined. Recombinant LSU1 specifically induced FSD2 enzyme activity, but not the activity of the non-interacting FSD1 (Fig. 4D). A similar trend was observed upon addition of GST–LSU2 but not GST alone or MBP fused to the unrelated NIM1-INTERACTING 1 (NIMIN1) (Supplementary Fig. S5D). Thus, mechanistically LSU1 and LSU2 can stimulate H_2_O_2_ production by direct interaction with and activation of FSD2.

### *Pseudomonas syringae* virulence effectors interfere with LSU1 functions

Stomata are a major entry point for bacterial pathogens, and of all LSU family members the guard cell-localized LSU1 preferentially interacted with bacterial virulence effectors in our Y2H-based network mapping experiments (Melotto *et al.*, 2006; Mukhtar *et al.*, 2011; Wessling *et al.*, 2014). H_2_O_2_ can trigger stomatal closure, but may also have signalling and defence functions (Desikan *et al.*, 2001; Van Aken and Whelan, 2012; de Torres Zabala *et al.*, 2015). Lastly, chloroplastic H_2_O_2_ production was recently shown to be an important element of PTI with which *Pst* virulence effectors can interfere (de Torres Zabala *et al.*, 2015). We therefore wondered if the LSU1–FSD2 interaction may be relevant in defence responses and if LSU-interacting bacterial effectors can interfere with it. First, we confirmed by Y2H the interaction of LSU1 with several bacterial virulence effectors (Fig. 5A), including the putatively chloroplast-localized *Pst* HopAT1, HopF3, and the C-terminal fragment of HopR1 (Mukhtar *et al.*, 2011; Wessling *et al.*, 2014; de Torres Zabala *et al.*, 2015). Using *in vitro* GST pull-down experiment, we tested if *Xanthomonas campestris* pv. *campestris* (*Xcc*) AvrXccC, *P. syringae* pv. *phaseolica* (*Psp*) AvrB2, and *Pst* HopR1 interfere with the LSU1–FSD2 interaction; the others were not tested for technical reasons. After pull-down of GST–FSD2, co-precipitation of LSU1 was assessed by western blot with antibodies to MBP, and band intensity was quantified (Fig. 5B). All three effectors effectively and specifically interfered with the interaction such that only 20–40% of MBP–LSU1 was recovered in the GST–FSD2 pull-down compared with controls (Fig. 5B, lower panel). Furthermore, microscopic analysis revealed that *Pst* infection during –S stress interfered with the chloroplastic localization of GFP–LSU1 such that the proportion of chloroplasts overlapping GFP fluorescence decreased from 73% to 41% and the PCC of the two fluorescence patterns decreased from 0.52 and 0.51 in untreated and mock-treated leaves, respectively, and to 0.09 in *Pst*-infected seedlings (Fig. 5C). Thus, virulence effectors interfere with several aspects of LSU1 function *in vitro* and *in vivo*.

### LSU proteins mediate resistance to *Pseudomonas syringae* infection under certain abiotic stress conditions

**Fig. 6.**
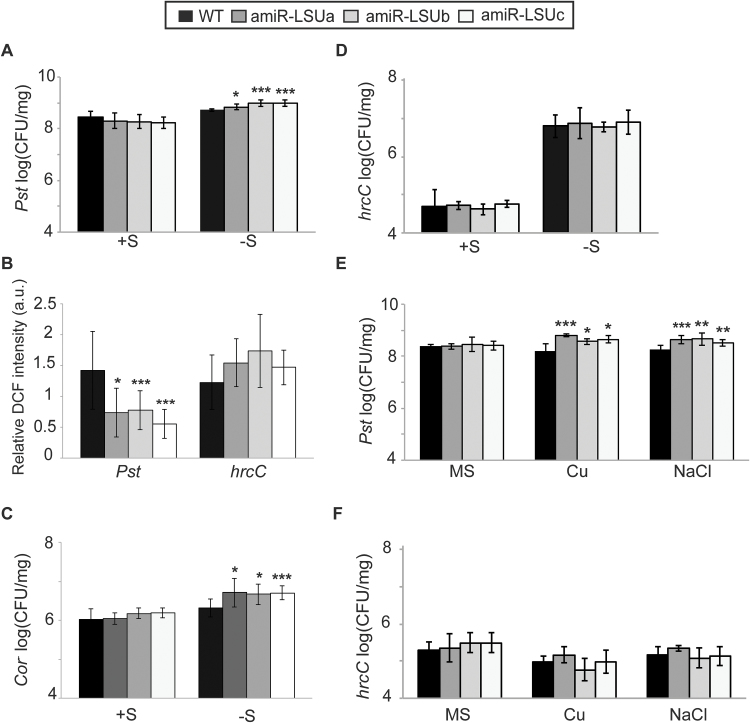
LSU proteins mediate defence responses during abiotic stress. WT and amiR-LSUa–c seedlings were flooded with bacterial suspension at 5 × 10^6^ CFU ml^–1^ of the *P. syringae* strains: *Pst* DC3000 (*Pst*), COR^–^, and *hrcC*. In (A) and (C–F), colony-forming units were determined 3 days post-infection (dpi) and normalized to fresh weight. Seedlings were grown in normal (+S) and –S conditions, or 1/2 MS conditions, or submitted to high Cu or NaCl, as indicated. (B) DCF fluorescence in guard cells of –S-grown lines at 1 dpi with the indicated bacterial strain normalized to mock treatment. Error bars correspond to the SD of at least three biologically independent experiments, and statistical differences from the WT were assessed using Student’s *t*-test and are indicated with asterisks (*P<0.05; **P<0.01; ***P<0.001).

These results led us to investigate genetically the immune function of LSU proteins, especially during abiotic stress. First we asked if *LSU* down-regulation alters plant susceptibility towards *Pst*. To perform infection assays under nutrient deficiency conditions, 12-day-old WT and amiR-LSUa–c seedlings grown *in vitro* on +S or –S were flooded with *Pst* suspensions and colony density was counted 3 days post-infection (dpi). As depicted on Fig. 6A, consistent with the lack of detectable LSU expression, no significant differences in pathogen susceptibility were found among lines under standard conditions (+S). However, growth of seedlings on –S resulted in mild but significant EDS in the amiR-LSU lines, as the colony count increased ~2-fold compared with the WT (Fig. 6A; Supplementary Table S1). Consistent with this phenotype and our findings above, the amiR-LSUa–c lines showed reduced ROS production in guard cell chloroplasts following *Pst* challenge 1 dpi (Fig. 6B).

Part of the *Pst* infectivity relies on the production of coronatine (COR), a jasmonic acid analogue that mediates stomatal re-opening and promotes bacterial virulence by suppressing salicylic acid-mediated defence responses (Cui *et al.*, 2005; Melotto *et al.*, 2006). Upon infection by the COR-defective mutant COR^–^, the amiR-LSUa–c lines still exhibit moderate EDS on –S (on average ~2.5 times higher than in the WT) (Fig. 6C). Thus, the EDS phenotype in the amiR-LSU lines is not part of the COR-induced responses. To test if this phenotype would be a component of PTI, the same assay was repeated using the Type III Secretion System (T3SS)-defective *hrcC* mutant, which elicits PTI but is unable to deliver effectors into the plant cytosol (Cunnac *et al.*, 2011) and therefore does not elicit ETI. The amiR-LSU lines did not exhibit an altered susceptibility towards *hrcC* in either growth condition (Fig. 6D), suggesting that the LSU-dependent immune function requires effector delivery or, formally, the T3SS itself. Consistently, infection of –S-stressed plants by *hrcC*, in contrast to WT *Pst*, did not cause an additionally increased ROS production in guard cell chloroplasts (Fig. 6B). Analysis of guard cell dynamics during initial phases of *Pst* infection further excluded impairments of *Pst*-triggered stomatal closure in amiR-LSU lines and revealed an altered pattern of stomatal dynamics in –S conditions. The WT and the amiR-LSUa–c lines effectively closed stomata 1 h post-infection (hpi) in +S and –S conditions, indicating that LSU proteins do not function during this stage of infection (Supplementary Fig. S6). However, in WT plants grown on –S media, stomata remained closed even at 4 hpi (Supplementary Fig. S6B). In amiR-LSUa and amiR-LSUc, stomata were ~20% and 30%, respectively, more open at 4 hpi compared with the WT (Supplementary Fig. S6B; Supplementary Table S3). Together, these observations reinforce the notion that in –S stress conditions, LSU proteins have a defence function that involves stimulating ROS production, but which is not mediated by COR and is not part of the *hrcC*-activated PTI. Consistent with this idea, the knock-out line *fsd2-2* also displayed a weak EDS phenotype under our S-deficient conditions (~1.5-fold more colonies than the WT; Supplementary Fig. S7).

Since LSU proteins are up-regulated in several stress conditions, we wondered if they might also have an immune function in abiotic stresses other than S deprivation. Indeed, amiR-LSU plants grown in the presence of high salt or Cu concentrations also exhibited moderate but significant EDS towards *Pst*, but not *hrcC*, demonstrating that LSU proteins support immune function in several abiotic stress conditions (Fig. 6D, E).

**Fig. 7.**
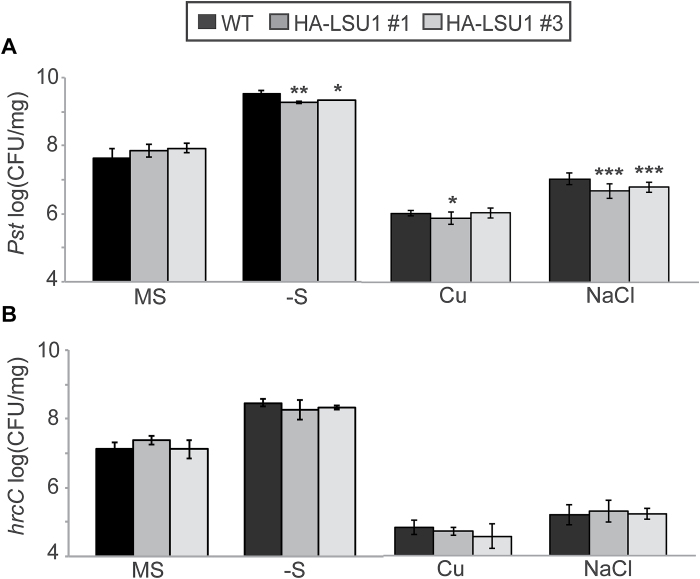
Ectopic expression of *LSU1* results in a moderate resistance to *P. syringae* during abiotic stress. The WT and two independent HA–LSU1 lines were grown and treated as in Fig. 5. Error bars correspond to the SD of at least three biologically independent experiments, and statistical differences from the WT were assessed using Student’s *t*-test and are indicated with asterisks (*P<0.05; **P<0.01; ***P<0.001).

To test if LSU overexpression results in complementary EDR, and to gather support for our suggestion that LSU1 might be responsible for the observed amiR-LSU phenotypes, we phenotypically characterized two independent *35S:HA-LSU1* overexpression lines using *Pst* and *hrcC*. A phenotypic pattern complementary to the amiR-LSUa–c lines was observed such that *LSU1* overexpression conferred significant EDR towards *Pst* during –S and high salt stress but had no effect under normal conditions or towards *hrcC* (Fig. 7; Supplementary Table S1).

## Discussion

In addition to understanding molecular plant response mechanisms to isolated abiotic and biotic stress, it is also important to characterize the integrated responses to combinatorial stress conditions. LSU proteins, which had only been implicated in –S stress, are highly connected hubs in a plant protein interactome network and intensely targeted by virulence effectors from evolutionarily distant pathogens (Arabidopsis Interactome Mapping Consortium, 2011; Mukhtar *et al.*, 2011; Wessling *et al.*, 2014). These observations motivated us to investigate the function of the encoded proteins to illuminate their normal physiological role and find out why effectors may have evolved to interfere with this function.

Our primary characterization of *LSU1* and *LSU2*, as representatives of this protein family, suggests a general function of these genes in abiotic stress responses. Both *LSU1* and *LSU2* transcripts are up-regulated in several abiotic stress conditions, and apparent post-translational regulation leads to protein accumulation in seedlings and, for LSU1, to stabilization of GFP–LSU1 in guard cells. Since all LSU family members have a serine-rich domain that includes putative phosphorylation sites (Supplementary Fig. S1A) and phosphorylation of LSU3 has been detected *in vivo* (Phosphat; http://phosphat.uni-hohenheim.de/), the impact of this post-translational modification on protein stability warrants further investigation.

The prominent localization of GFP–LSU1 in guard cells, reinforced by the *LSU1* down-regulation in cotyledons of the stomataless mutants *spch-3* and *mute-3* (de Marcos *et al.*, 2015), prompted us to investigate potential functions of LSU in stomatal dynamics. Indeed, transgenic lines with reduced *LSU* levels display impaired stomatal closure under –S. Moreover, the amiR-LSU lines exhibited profoundly reduced ROS production in guard cells when exposed to stress conditions that induce LSU transcription, such as high salinity and –S. This suggests that LSU proteins, most proobably LSU1, promote stomatal closure in response to abiotic stress by stimulating chloroplastic ROS production. In guard cells, ROS levels depend on a complex interplay between ABA and ethylene whereby ethylene negatively regulates ABA-dependent stomatal closure by alleviating ROS levels, while, conversely, ABA represses ethylene production (Tanaka *et al.*, 2005, 2006; Watkins *et al.*, 2014). However, upon exogenous ABA treatment, stomata of amiR-LSU lines closed normally, indicating that the ABA-triggered stomatal closure is not impaired. It is possible that, similarly to ethylene, LSUs function upstream of ABA. This appears unlikely given that S limitation negatively impacts ethylene biosynthesis due to reduced methionine availability (Bürstenbinder *et al.*, 2007) and also leads to drastically reduced ABA levels (Cao *et al.*, 2014). We therefore hypothesize that LSU proteins may participate in an alternative mechanism to trigger stomatal closure in some stress conditions. The exclusive presence of LSU proteins among gymno- and angiosperms provides evolutionary support for the proposed role of LSU proteins in guard cells.

A hint towards a possible mechanism by which LSUs induce ROS production came from our Arabidopsis interactome map, where LSU proteins were found to interact with FSD2 (Arabidopsis Interactome Mapping Consortium, 2011). We demonstrate here that LSU1 and LSU2 specifically interact with FSD2 *in vitro* and *in planta*, and stimulate its enzyme activity. Consistent with the idea that LSU1 activates FSD2 *in planta*, GFP–LSU1, but not GFP–LSU2, was found in guard cell patterns that largely overlap with chloroplast autofluorescence, and upon S deprivation amiR-LSU lines exhibit reduced H_2_O_2_ production in guard cells. The increased H_2_O_2_ levels can trigger stomatal closure and potentially other effects, such as transcriptional regulation (Vandenabeele *et al.*, 2003; Van Aken and Whelan, 2012) (Supplementary Fig. S8). A heterodimeric complex of FSD2/FSD3 was previously shown to protect chloroplast nucleoids from ROS (Myouga *et al.*, 2008). This study found that FSD3 localized exclusively to speckles in the nucleoids, while, consistent with our data, FSD2 exhibited a wider chloroplastic localization suggesting additional functions (Myouga *et al.*, 2008). We propose that abiotic stress conditions lead to stabilization of LSU1, especially in guard cells, where it enters chloroplasts and stimulates ROS production by activating FSD2. The question of how LSU1, despite the lack of transit peptide, is imported to the chloroplast remains to be answered, though examples of chloroplast- and nuclear-localized proteins using non-canonical importing signals or interacting partners have been reported (Miras *et al.*, 2002, 2007; Withers *et al.*, 2012). Biochemically, it is likely that FSD2 activation by LSU1 represents another example of factor-associated SOD activation, which has been previously reported for all SOD isoforms (Luk *et al.*, 2003; Chu *et al.*, 2005; Kuo *et al.*, 2013). The capacity of LSU to stimulate FSD2, the small size, and lack of clearly defined globular domains moreover beg the question of whether LSU proteins might also have chaperone-like activity for other clients.

Besides their function for gas exchange, stomata are natural orifices that constitute entry points for bacterial pathogens into the plant apoplast (Melotto *et al.*, 2006). Although plants reinforce stomatal immunity upon bacterial recognition during PTI, bacterial pathogens such as *Pst* can counteract this effect by producing COR and virulence effectors to re-open stomata or prevent stomatal closure, respectively (Melotto *et al.*, 2006; Gudesblat *et al.*, 2009). Given that we identified LSU proteins as targets of virulence effectors, we hypothesized that LSU-mediated stomatal closure could also have a defence function with which pathogens may aim to interfere. In support of this, we found that amiR-LSU lines do exhibit an EDS phenotype in conditions of abiotic stress whereas LSU1 overexpression lines display complementary EDR phenotypes. Upon infection by *hrcC*, this phenotype was not detectable, whereas it was unaltered upon infection by a COR^–^ strain, indicating that the LSU-dependent immune function is not due to a general LSU function in these conditions and not due to PTI or COR-triggered pathways. Abrogation of *LSU* expression did not directly impair stomatal closure in response to *Pst* infection during S scarcity; however, it caused a moderate and faster stomatal re-opening in comparison with the WT at 4 hpi. The weak immune effects may be due to the inability of the amiR-LSU lines to maintain closed stomata during the early phase of the infection, but independent mechanisms cannot be excluded and should be further investigated.

Chloroplastic ROS were recently shown to be an important defence signal that *Pst* can suppress by only partly known mechanisms (de Torres Zabala *et al.*, 2015). Here, we demonstrated that *Pst* effectors can interfere with the activating interaction of LSU1 with FSD2. In addition, *Pst* infection prevents GFP–LSU1 from entering guard cell chloroplasts, and our genetic data confirm the immune function of LSU proteins during high salinity or –S stress. In these conditions, artificially reduced *LSU* levels abolish the normally increased ROS production in response to *Pst* infection and cause a modest EDS phenotype. Lack of FSD2 activity in the *fsd2*-2 line resulted in a similar degree of *Pst* susceptibility, whereas ectopic expression of *LSU1* results in modest EDR phenotypes.

Recent studies already implicated virulence effectors in modulating stomatal function by demonstrating that *Pst* HopF2 and HopM1 can suppress stomatal immune responses and ROS burst *in planta* during bacterial infection (Hurley *et al.*, 2014; Lozano-Duran *et al.*, 2014). Thus, we suggest that upon infection with virulent *Pst*, the antagonistic interplay of effectors triggering weak ETI (Gassmann, 2005; Fabro *et al.*, 2011) and effectors interfering with LSU results in an overall small, but significant immune phenotype in amiR-LSU lines, demonstrating the immune function of LSU proteins in plants experiencing –S or salt stress (Supplementary Fig. S8).

Taken together, we demonstrate that LSU proteins stimulate production of H_2_O_2_ and potentially other ROS, and effect stomatal closure in response to several types of abiotic stress, including high salinity or S depletion. Mechanistically, this stimulation is at least in part achieved via LSU up-regulation and subsequent interaction with and activation of the SOD FSD2. Moreover, we show that LSU proteins are elements of the plant immune response when plants experience simultaneous abiotic stress.

## Author contributions

experiments and figures: AG-M. Assistance with Y2H experiments: MA and AA. Design of experiments: AG-M, PE, JLD, and PB. Manuscript writing: AG-M and PB. Critical manuscript reading and editing: PE and JLD. All authors discussed and commented on the article.

## Supplementary Material

Supplementary Data
