# Supplementary material for: LSU network hubs integrate abiotic and biotic stress responses via interaction with the superoxide dismutase FSD2
Source: J Exp Bot. 2017 Feb 16;68(5):1185–97. doi: 10.1093/jxb/erw498 (PMC5441861; doi:10.1093/jxb/erw498)
Supplement: Supplementary Data [file erw498_Supplementary_Data.zip › supplementary_figures_S1_S8_Tables_S1_S3.pdf]

Supplementary Figures

Figure S1

A

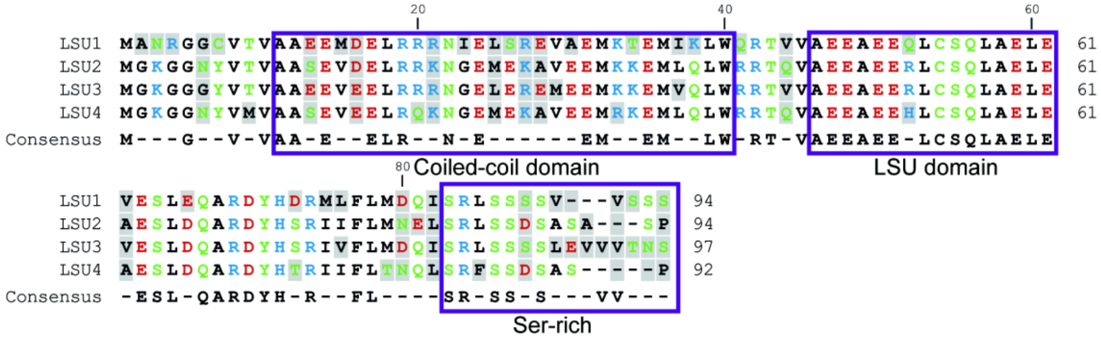

B

	LSU2	LSU4	LSU3	LSU1
LSU2	100	90.22	77.66	66.30
LSU4		100	73.96	60.44
LSU3			100	76.60
LSU1				100

C

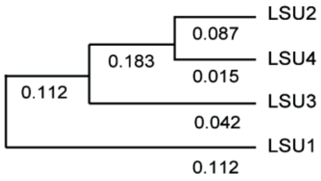

Fig. S1. Primary sequence analysis and additional characterisation of LSU proteins. A) Multiple sequence alignment of Arabidopsis LSU1-4 proteins using ClustalW. Similar residues are highlighted with same colours, while divergent amino acids are shaded in grey. Lila boxes highlight three conserved features in LSU proteins: (i) a predicted coiled-coil domain, (ii) a central domain exclusively conserved on LSU proteins that we denoted LSU domain and (iii) a serine-rich region. Relative positions and consensus sequence are provided. **B)** Matrix representing the percentage of sequence similarity among LSU1-4. **C)** Dendogram of the Arabidopsis LSU proteins using Neighbour Joining and clustering. Protein distance measure is indicated according to Jukes-Cantor.

Figure S2

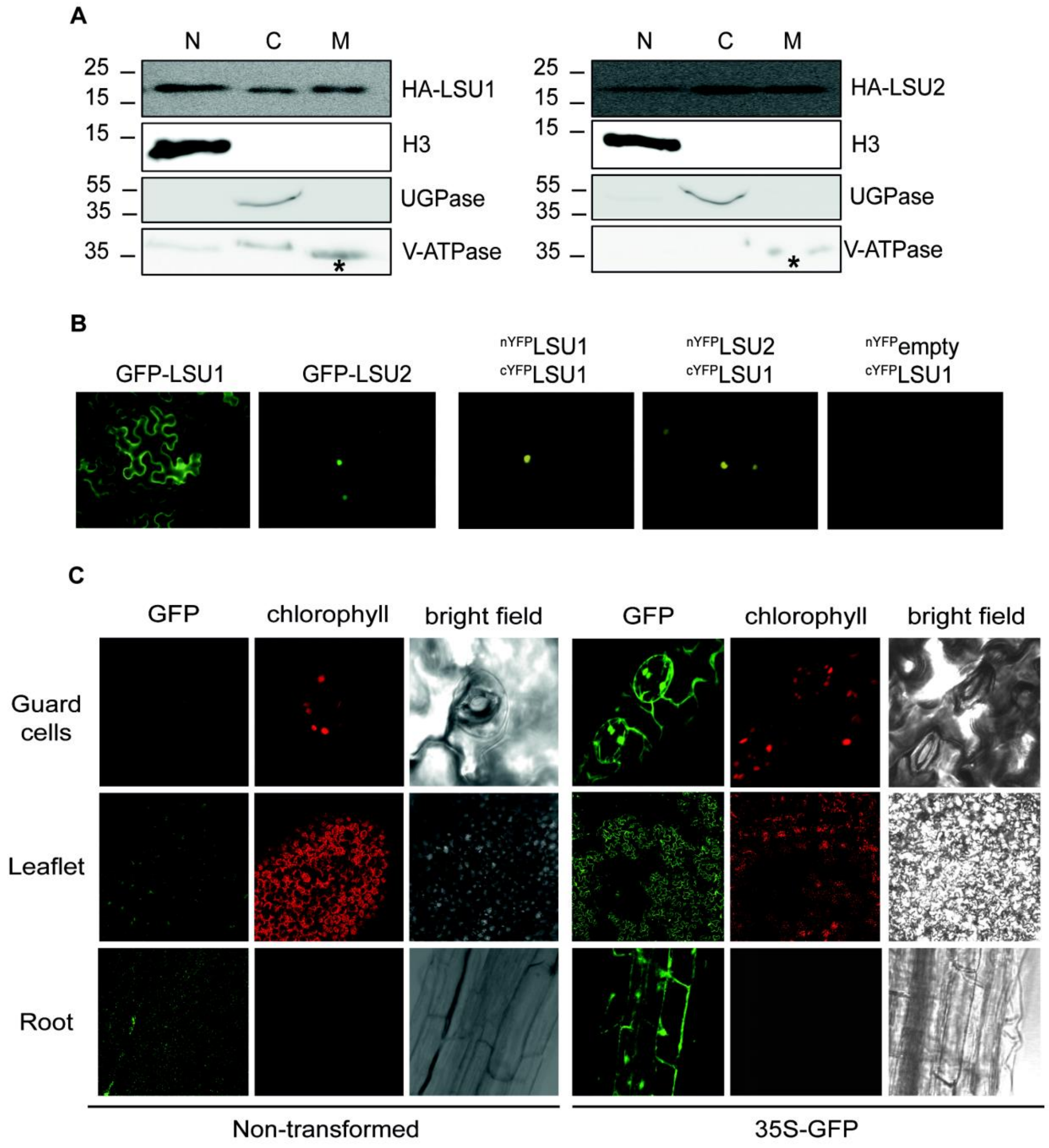

Fig. S2. Additional characterisation of amiR-LSU lines. A) Biochemical fractionation of HA-LSU1 and HA-LSU2. Enriched nuclear (N), cytosolic (C) and microsomal (M) fractions obtained from *HA-LSU1* and *HA-LSU2* seedlings grown on –S were assayed by Western blot using antibodies to HA or organelle markers HISTONE3 (H3); UDP-GLUOSE PYROPHOSPHORYLASE (UGPase) and VACUOLAR ATPASE (V-ATPase; specific band indicated with an asterisk). Relative molecular weight is indicated in kDa. **B**) Subcellular localisation of LSU1 and LSU2 on *Nicotiana benthamiana*. Representative fluorescent microscopy pictures of *N. benthamiana* epidermal leaves transiently expressing GFP-LSU1

and GFP-LSU2 (left panel) or co-injected with LSU1 and LSU2 for Bimolecular Fluorescent Complementation to assess LSU1 homo- and heterodimerisation (right panel). Expression with an empty vector is provided as negative control. C) Confocal imaging of non-transformed Arabidopsis and *35SCaMV-GFP* lines using similar settings as for the Figure 1C.

Figure S3

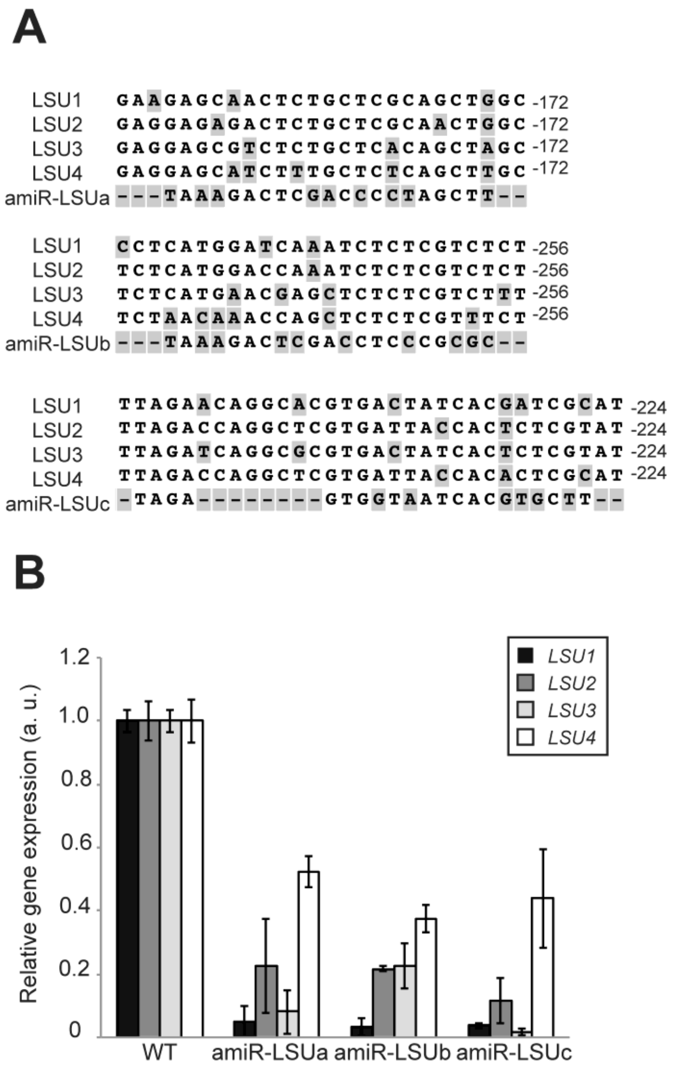

Fig. S3. Generation of amiR-LSU lines. A) Sequences selected to generate amiRNA lines targeting *LSU* genes. Multiple sequence alignment of *LSU1-4* coding sequences and amiR-LSUa-c. Non-matching residues are shaded in grey and the relative position of the sequence is indicated. **B)** amiR-LSU lines reduce *LSU1-4* gene expression. *LSU1-4* expression levels were determined on seedlings corresponding to WT and the amiR-LSUa-c lines grown on –S by qPCR using specific oligonucleotides.

Figure S4

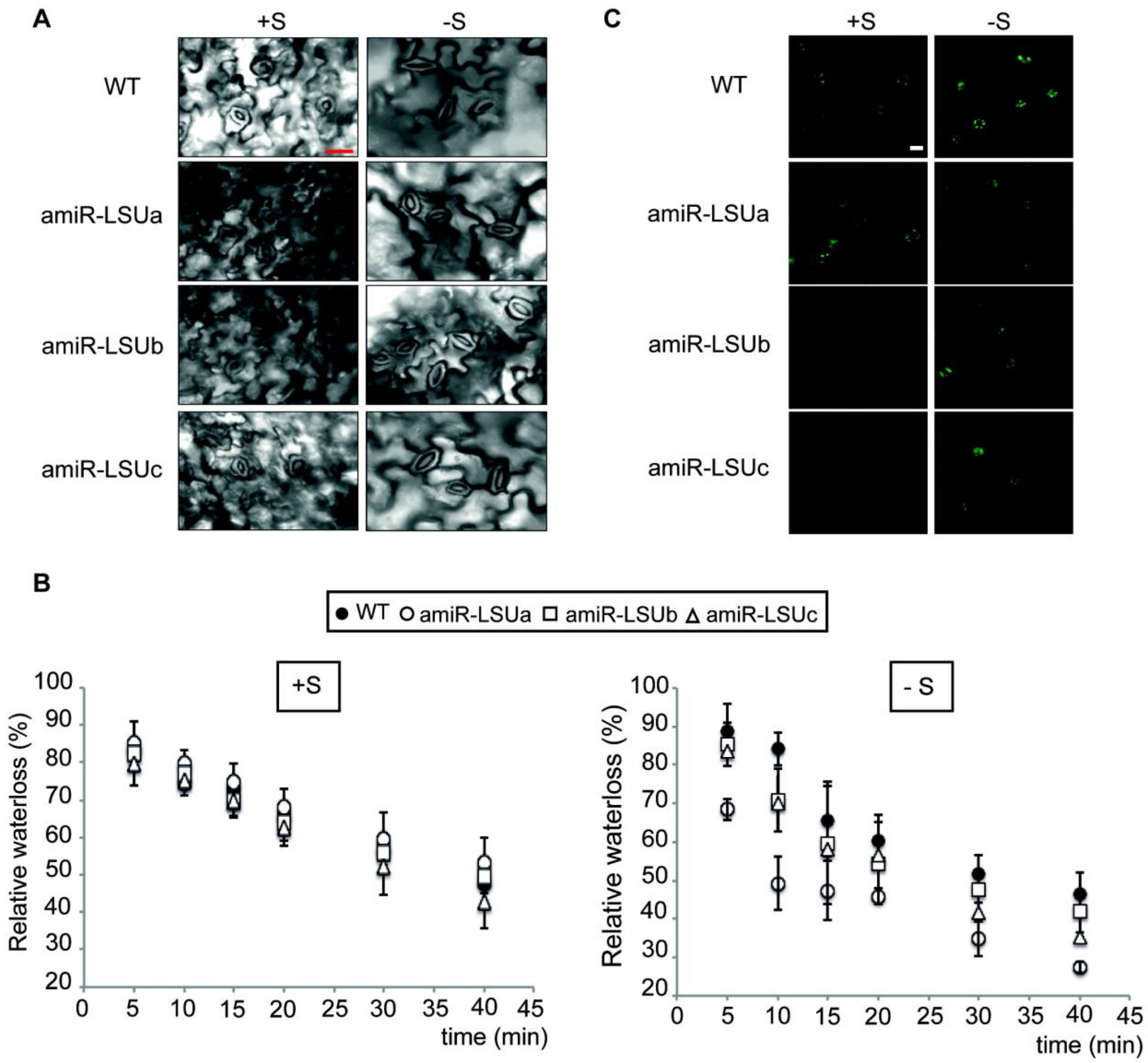

Fig. S4. Additional guard cell phenotypes of amiR-LSU lines. A) Suppression of LSU expression constrains stomata closure in –S. Representative images of guard cells corresponding WT and amiR-LSU lines grown on standard conditions or –S (scale bar 25 μm) **B)** amiR-LSUa-c display increased water loss. Fresh weight of groups of 12-d-old seedlings grown on +S or –S was recorded and normalised to 100%. Water loss was estimated as loss of fresh weight over time after removing seedlings from plates. Error bars correspond to standard deviation of 4 batches of seedlings in a representative experiment. **C)** ROS production in guard cells is impaired in amiR-LSU lines during S deficiency conditions. Representative pictures of abaxial guard cells of WT and amiR-LSUa-c seedlings grown under +S or –S showing DCF fluorescence intensity (scale bar 25 μm) are provided.

Figure S5

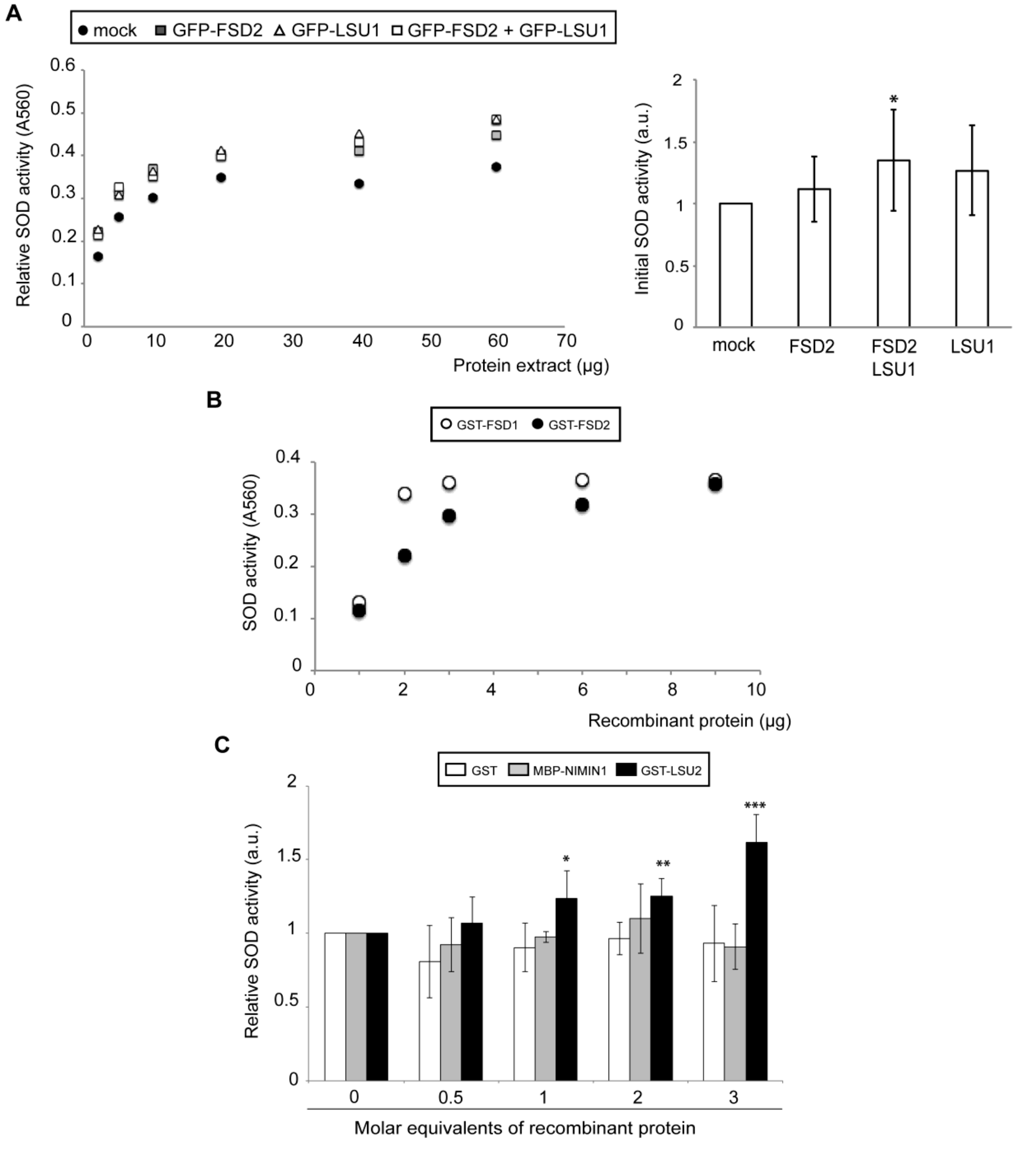

Fig. S5. Determination of FSD activity *in vivo* and *in vitro*. A) LSU1 enhances FSD2 activity *in vivo*. SOD activity was spectrophotometrically determined with increasing protein extracts from *N. benthamiana* leaves agro-injected with mock, GFP-LSU1, GFP-FSD2 or a combination of both. The initial activity is represented in the right panel. Error bars correspond to standard deviation of 4 biologically independent measurements. **B)** Recombinant GST-FSD1 and GST-FSD2 proteins purified from *E. coli* are enzymatically active. SOD activity of increasing concentrations of recombinant GST-FSD1 or GST-FSD2

was spectrophotometrically determined and represented. **C)** Relative *in vitro* SOD activity of recombinant GST-FSD2 in the presence of increasing concentrations of the indicated recombinant proteins. Error bars correspond to the standard deviation of 3 independent experiments. In all cases significant differences to control conditions were assessed using Student's t-test and indicated by asterisks (* $P < 0.05$; ** $P < 0.01$; *** $P < 0.001$).

Figure S6

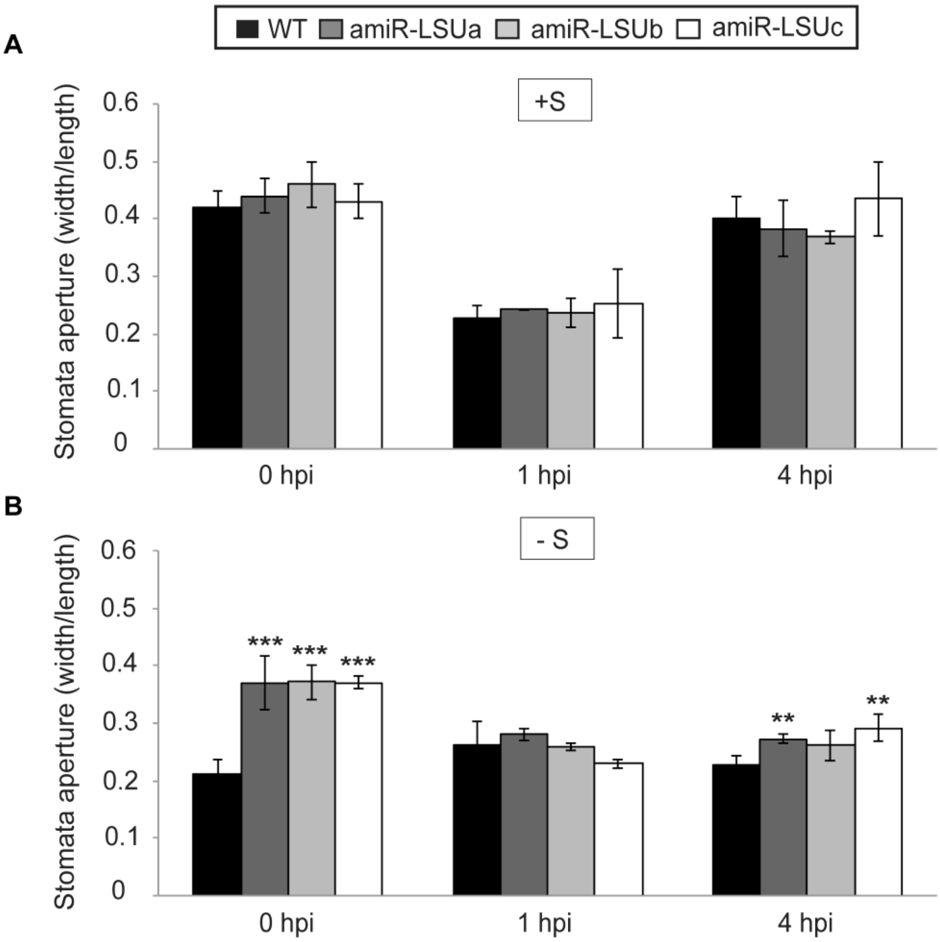

Fig. S6. Stomata dynamics during initial phases of *P. syringae* infection. Stomata aperture was determined as the ratio of ostiol width to length in 12-d-old WT and amiR-LSU seedlings grown in normal (+S, **A**) or –S conditions (**B**) after the indicated hours post-infection (hpi) with *Pst*. Error bars correspond to the standard deviation of 3 independent experiments. In all cases significant differences to WT were assessed using Student's t-test and indicated by asterisks (** $P < 0.01$; *** $P < 0.001$).

Figure S7

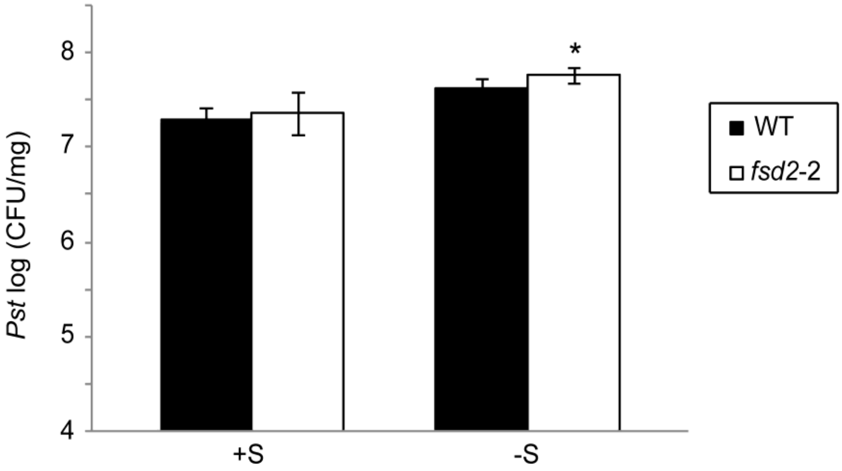

Fig. S7. *fsd2*-2 displays a moderate enhanced disease susceptibility to *P. syringae* during sulphur deficiency. 12-d-old WT and the *fsd2*-2 seedlings cultivated as indicated were challenged with *P. syringae* and the colony counts determined. Error bars correspond to the standard deviation of 3 independent experiments and significant differences to WT according to Student's t-test indicated by asterisks (* $P < 0.05$).

Figure S8

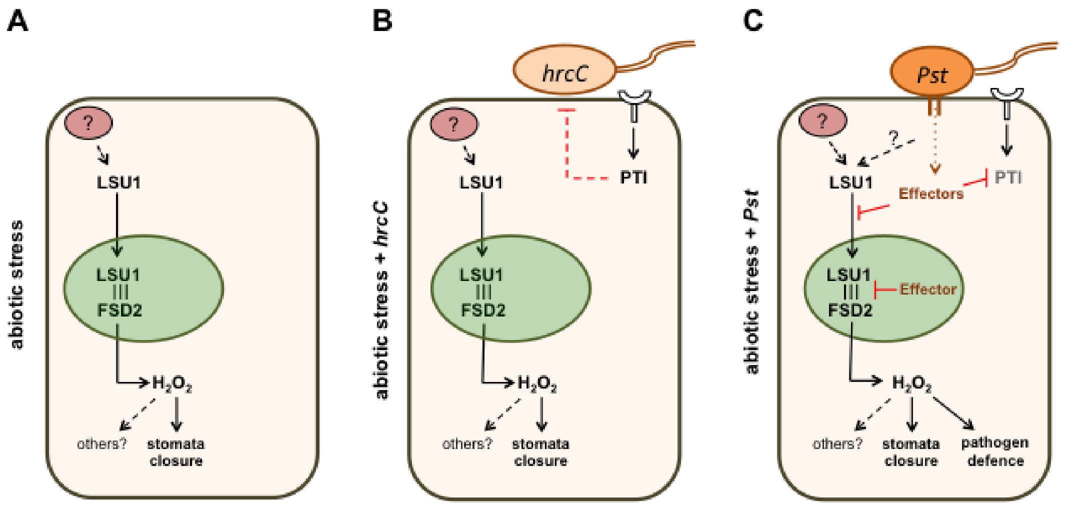

Fig. S8. A model for LSU1 function in guard cells during combined abiotic and biotic stress. A) In abiotic stress conditions LSU1 is induced and stabilised, localises to guard cell chloroplasts and stimulates H_2O_2 production via interaction with and activation of FSD2. The resulting increase in H_2O_2 promotes stomata closure and may trigger other effects such as transcriptional changes. **B)** Upon infection by the T3SS defective *hrcC*, which only activates PTI, LSU1 does not contribute to pathogen defence in normal nor in stress conditions. **C)** Upon infection by *Pst* the defence function of LSU1 is activated by a currently unknown mechanism. Although *Pst* virulence effectors interfere with different aspect of LSU1 function, this interference is incomplete and LSU1 further stimulates H_2O_2 production and contributes to pathogen defence in conditions of abiotic stress. Neither abiotic stress nor infection by pathogens alone is sufficient to activate this defence function of LSU1.

Supplementary Tables

Table S1. Statistic analysis of experiments in the present work. Average, standard deviation, number of replicates and p-value according to Student's t-test for all graphs and statistical analyses in this study.

Fig. 1A

condition	gene	average	sd	p-value	n
minus S	*LSU1*	11,86	2,70	0,00	3
minus Fe	*LSU1*	2,89	0,86	0,02	3
Cu	*LSU1*	7,26	2,48	0,01	3
DTT	*LSU1*	1,34	0,91	0,19	3
NaCl	*LSU1*	3,53	1,27	0,01	3
pH 8	*LSU1*	16,73	8,42	0,02	3
minus S	*LSU2*	3,47	1,96	0,04	3
minus Fe	*LSU2*	1,75	0,58	0,03	3
high Cu	*LSU2*	3,40	1,26	0,01	3
DTT	*LSU2*	1,18	0,56	0,20	3
NaCl	*LSU2*	4,98	2,45	0,02	3
pH 8	*LSU2*	5,66	2,78	0,02	3

Fig. 2

genotype	condition	average	sd	p-value	n
WT	plus S	0,43	0,03		3
amiR-LSUa	plus S	0,44	0,03		3
amiR-LSUb	plus S	0,46	0,04		3
amiR-LSUc	plus S	0,43	0,03		3
WT	minus S	0,30	0,02		3
amiR-LSUa	minus S	0,43	0,03	0,00	3
amiR-LSUb	minus S	0,45	0,02	0,00	3
amiR-LSUc	minus S	0,45	0,00	0,00	3
WT	plus S + ABA	0,28	0,02		3
amiR-LSUa	plus S + ABA	0,28	0,01	0,07	3
amiR-LSUb	plus S + ABA	0,26	0,04	0,00	3
amiR-LSUc	plus S + ABA	0,29	0,03	0,01	3
WT	minus S + ABA	0,28	0,02		3
amiR-LSUa	minus S + ABA	0,28	0,02		3
amiR-LSUb	minus S + ABA	0,28	0,01		3
amiR-LSUc	minus S + ABA	0,29	0,02		3

Fig. 3B

genotype	condition	average	sd	p-value	n	amiR/WT
WT	plus S	61,04	11,31		3	
amiR-LSUa	plus S	79,64	7,46	0,01	3	1,30
amiR-LSUb	plus S	67,47	15,11	0,23	3	1,11
amiR-LSUc	plus S	49,63	10,81	0,06	3	0,81
WT	minus S	100,00	0,00		8	
amiR-LSUa	minus S	65,14	10,48	0,00	3	0,65
amiR-LSUb	minus S	55,25	19,85	0,01	3	0,55
amiR-LSUc	minus S	67,06	4,27	0,00	4	0,67

Fig. 3C

genotype	condition	average	sd	p-value	n	amiR/WT
WT	minus S	103,32	8,76		4	
amiR-LSUa	minus S	70,48	14,01	0,00	4	0,68
amiR-LSUb	minus S	72,63	15,72	0,01	4	0,70
amiR-LSUc	minus S	71,98	28,18	0,04	4	0,70

Fig. 3D

genotype	condition	average	sd	p-value	n	amiR/WT
WT	plus Cu	100,00	26,03		22	
amiR-LSUa	plus Cu	60,40	18,05	0,00	22	0,60
amiR-LSUb	plus Cu	47,66	23,11	0,00	10	0,48
amiR-LSUc	plus Cu	49,50	12,18	0,00	11	0,50
WT	plus NaCl	100,00	34,22		19	
amiR-LSUa	plus NaCl	63,98	24,31	0,02	5	0,64
amiR-LSUb	plus NaCl	50,26	26,67	0,00	9	0,50
amiR-LSUc	plus NaCl	47,31	21,43	0,00	11	0,81

Fig. 4D

Recomb prot	equivalent LSU1	average	sd	p-value	n
GST-FSD1	0	1	0		3
	0,5	0,91	0,65	0,09	3
	1	1,03	0,50	0,05	3
	3	1,85	1,21	0,00	3
GST-FSD2	0	1	0		3
	0,5	3,07	2,16	0,42	3
	1	5,07	3,32	0,62	3
	3	10,80	2,10	0,69	3

Fig. 5B

competitor	ratio	average	sd	p-value	n
-	LSU1/LSU1 control	1,00	0,00		3
NIMIN1	LSU1/LSU1 control	1,01	0,40	0,40	3
avrB2	LSU1/LSU1 control	0,20	0,08	0,00	3
avrC	LSU1/LSU1 control	0,46	0,18	0,00	3
HopR1	LSU1/LSU1 control	0,36	0,22	0,00	3

Fig. 5C

genotype	condition	average	sd	p-value	n
GFP-LSU1	mock	0,51	0,13		10
GFP-LSU1	DC3000	0,09	0,09	0,00	14

Fig. 6A

genotype	condition	average	sd	p-value	n	amir/WT
WT	DC3000 plus S	8,46	0,22		5	
amiR-LSUa	DC3000 plus S	8,31	0,30	0,20	5	0,71
amiR-LSUb	DC3000 plus S	8,27	0,26	0,13	5	0,65
amiR-LSUc	DC3000 plus S	8,23	0,24	0,08	5	0,59
WT	DC3000 minus S	8,72	0,05		4	
amiR-LSUa	DC3000 minus S	8,84	0,11	0,04	4	1,32
amiR-LSUb	DC3000 minus S	8,99	0,13	0,00	5	1,86
amiR-LSUc	DC3000 minus S	8,99	0,13	0,00	5	1,86

Fig. 6B

genotype	condition	average	sd	p-value	n	amiR/ WT
WT	DC3000/mock	1,42	0,63		11	
amiR-LSUa	DC3000/mock	0,74	0,39	0,05	3	0,52
amiR-LSUb	DC3000/mock	0,77	0,31	0,00	12	0,54
amiR-LSUc	DC3000/mock	0,56	0,24	0,00	7	0,39
WT	hrcC/mock	1,23	0,44		11	
amiR-LSUa	hrcC/mock	1,54	0,39	0,08	6	1,26
amiR-LSUb	hrcC/mock	1,74	0,59	0,09	5	1,42
amiR-LSUc	hrcC/mock	1,47	0,28	0,16	6	1,20

Fig. 6C

genotype	condition	average	sd	p-value	n	amir/WT
WT	COR^- plus S	6,01	0,27		3	
amiR-LSUa	COR^- plus S	6,04	0,15	0,43	3	1,08
amiR-LSUb	COR^- plus S	6,18	0,13	0,20	3	1,48
amiR-LSUc	COR^- plus S	6,19	0,13	0,18	3	1,51
WT	COR^- minus S	6,31	0,23		4	
amiR-LSUa	COR^- minus S	6,71	0,37	0,05	5	2,51
amiR-LSUb	COR^- minus S	6,63	0,27	0,03	6	2,09
amiR-LSUc	COR^- minus S	6,70	0,18	0,00	6	2,45

Fig. 6D

genotype	condition	average	sd	p-value	n	amir/WT
WT	hrcC plus S	4,69	0,44		4	
amiR-LSUa	hrcC plus S	4,71	0,10	0,46	4	1,05
amiR-LSUb	hrcC plus S	4,62	0,14	0,38	4	0,85
amiR-LSUc	hrcC plus S	4,76	0,09	0,39	4	1,17
WT	hrcC minus S	6,80	0,30		4	
amiR-LSUa	hrcC minus S	6,89	0,41	0,39	3	1,23
amiR-LSUb	hrcC minus S	6,78	0,13	0,48	3	0,95
amiR-LSUc	hrcC minus S	6,91	0,32	0,49	3	1,29

Fig. 6E

genotype	condition	average	sd	p-value	n	amir/WT
WT	DC3000 MS	8,40	0,05		4	
amiR-LSUa	DC3000 MS	8,38	0,11	0,38	4	0,95
amiR-LSUb	DC3000 MS	8,45	0,27	0,31	4	1,12
amiR-LSUc	DC3000 MS	8,41	0,17	0,43	4	1,02
WT	DC3000 plus Cu	8,20	0,27		4	
amiR-LSUa	DC3000 plus Cu	8,80	0,05	0,00	4	3,98
amiR-LSUb	DC3000 plus Cu	8,56	0,12	0,03	4	2,29
amiR-LSUc	DC3000 plus Cu	8,65	0,14	0,02	4	2,81
WT	DC3000 plus NaCl	8,27	0,15		5	
amiR-LSUa	DC3000 plus NaCl	8,64	0,17	0,00	5	2,34
amiR-LSUb	DC3000 plus NaCl	8,66	0,24	0,01	5	2,45
amiR-LSUc	DC3000 plus NaCl	8,51	0,12	0,01	5	1,73

Fig. 6F

genotype	condition	average	sd	p-value	n	amir/WT
WT	hrcC MS	5,31	0,22		5	
amiR-LSUa	hrcC MS	5,35	0,38	0,42	5	1,09
amiR-LSUb	hrcC MS	5,50	0,27	0,14	5	1,55
amiR-LSUc	hrcC MS	5,49	0,27	0,12	5	1,51
WT	hrcC plus Cu	4,98	0,16		5	
amiR-LSUa	hrcC plus Cu	5,17	0,21	0,06	5	1,55
amiR-LSUb	hrcC plus Cu	4,77	0,31	0,09	5	0,61
amiR-LSUc	hrcC plus Cu	4,98	0,30	0,49	5	1
WT	hrcC plus NaCl	5,16	0,24		5	
amiR-LSUa	hrcC plus NaCl	5,35	0,08	0,04	5	1,55
amiR-LSUb	hrcC plus NaCl	5,09	0,27	0,34	5	0,85
amiR-LSUc	hrcC plus NaCl	5,14	0,26	0,45	5	0,95

Fig. 7A

genotype	condition	average	sd	p-value	n	HA-LSU1/WT
WT	DC3000 plus S	7,64	0,28		3	
HA-LSU1 #1	DC3000 plus S	7,86	0,19	0,16	3	1,65
HA-LSU1 #3	DC3000 plus S	7,93	0,13	0,09	3	1,94
WT	DC3000 minus S	9,52	0,09		3	
HA-LSU1 #1	DC3000 minus S	9,28	0,04	0,01	3	0,57
HA-LSU1 #3	DC3000 minus S	9,35	0,07	0,05	3	0,67
WT	DC3000 plus Cu	5,86	0,08		8	
HA-LSU1 #1	DC3000 plus Cu	5,73	0,16	0,03	8	0,74
HA-LSU1 #3	DC3000 plus Cu	5,86	0,13	0,48	8	1
WT	DC3000 plus NaCl	6,79	0,15		7	
HA-LSU1 #1	DC3000 plus NaCl	6,46	0,20	0,00	8	0,47
HA-LSU1 #3	DC3000 plus NaCl	6,56	0,14	0,01	8	0,59

Fig. 7B

genotype	condition	average	sd	p-value	n	stress/MS
WT	hrcC plus S	7,14	0,19		3	
HA-LSU1 #1	hrcC plus S	7,37	0,13	0,07	3	1,69
HA-LSU1 #3	hrcC plus S	7,11	0,27	0,46	3	0,93
WT	hrcC minus S	8,47	0,10		3	
HA-LSU1 #1	hrcC minus S	8,26	0,28	0,15	3	0,62
HA-LSU1 #3	hrcC minus S	8,33	0,06	0,07	3	0,72
WT	hrcC plus Cu	4,65	0,16		6	
HA-LSU1 #1	hrcC plus Cu	4,57	0,10	0,14	6	0,83
HA-LSU1 #3	hrcC plus Cu	4,45	0,29	0,06	6	0,63
WT	hrcC plus NaCl	4,94	0,24		8	
HA-LSU1 #1	hrcC plus NaCl	5,03	0,26	0,26	6	1,23
HA-LSU1 #3	hrcC plus NaCl	4,97	0,13	0,39	6	1,07

Fig. S3B

genotype	condition	gene	average	sd	n
WT	minus S	*LSU1*	1,00	0,04	3
	minus S	*LSU2*	1,00	0,06	3
	minus S	*LSU3*	1,00	0,04	3
	minus S	*LSU4*	1,00	0,07	3
amiR-LSUa	minus S	*LSU1*	0,05	0,05	3
	minus S	*LSU2*	0,22	0,15	3
	minus S	*LSU3*	0,08	0,07	3
	minus S	*LSU4*	0,52	0,05	3
amiR-LSUb	minus S	*LSU1*	0,03	0,03	3
	minus S	*LSU2*	0,22	0,01	3
	minus S	*LSU3*	0,23	0,07	3
	minus S	*LSU4*	0,38	0,04	3
amiR-LSUc	minus S	*LSU1*	0,04	0,01	3
	minus S	*LSU2*	0,12	0,07	3
	minus S	*LSU3*	0,02	0,01	3
	minus S	*LSU4*	0,44	0,16	3

Fig. S4B

genotype	condition	average	sd	p-value	n
WT	+S 0 min	100,00	0,00		4
	+S 5 min	83,12	5,85		4
	+S 10 min	74,68	9,49		4
	+S 15 min	72,99	9,83		4
	+S 20 min	65,80	8,95		4
	+S 30 min	56,87	10,39		4

	+S 40 min	47,73	13,97		4
amiR-LSUa	+S 0 min	100,00	0,00		4
	+S 5 min	85,02	3,49	0,30	4
	+S 10 min	79,62	3,32	0,18	4
	+S 15 min	74,97	2,47	0,35	4
	+S 20 min	68,18	5,46	0,33	4
	+S 30 min	59,40	5,50	0,34	4
	+S 40 min	53,19	3,76	0,24	4
amiR-LSUb	+S 0 min	100,00	0,00		4
	+S 5 min	82,63	1,17	0,44	4
	+S 10 min	76,89	1,74	0,33	4
	+S 15 min	69,99	4,30	0,30	4
	+S 20 min	63,91	4,77	0,36	4
	+S 30 min	55,77	3,40	0,42	4
	+S 40 min	49,54	4,54	0,41	4
amiR-LSUc	+S 0 min	100,00	0,00		4
	+S 5 min	79,77	5,87	0,23	4
	+S 10 min	75,04	3,82	0,47	4
	+S 15 min	69,94	4,66	0,30	4
	+S 20 min	62,70	4,90	0,28	4
	+S 30 min	52,14	7,44	0,24	4
	+S 40 min	42,63	6,87	0,27	4
WT	-S 0 min	100,00	0,00		4
	-S 5 min	88,88	6,94		4
	-S 10 min	84,24	4,33		4
	-S 15 min	65,38	9,15		4
	-S 20 min	60,46	6,66		4
	-S 30 min	51,74	4,82		4
	-S 40 min	46,30	5,93		4
amiR-LSUa	-S 0 min	100,00	0,00		4
	-S 5 min	68,38	10,79	0,01	4
	-S 10 min	49,26	7,85	0,00	4
	-S 15 min	47,34	5,87	0,01	4
	-S 20 min	45,80	5,89	0,01	4
	-S 30 min	34,78	3,83	0,00	4
	-S 40 min	27,42	8,18	0,00	4
amiR-LSUb	-S 0 min	100,00	0,00		4
	-S 5 min	85,30	5,61	0,01	4
	-S 10 min	70,90	8,23	0,00	4
	-S 15 min	59,60	15,92	0,14	4

	-S 20 min	54,50	8,08	0,06	4
	-S 30 min	47,60	3,49	0,01	4
	-S 40 min	41,90	5,37	0,00	4
amiR-LSUc	-S 0 min	100,00	0,00		4
	-S 5 min	83,36	2,68	0,09	4
	-S 10 min	69,94	7,10	0,01	4
	-S 15 min	58,07	7,78	0,13	4
	-S 20 min	56,64	2,08	0,20	4
	-S 30 min	41,48	4,50	0,02	4
	-S 40 min	35,16	1,41	0,01	4

Fig. S5A

combination		average	sd	p-value	n
mock		1,00	0,00		4
LSU1		1,11	0,26	0,18	4
LSU1 + FSD2		1,35	0,41	0,05	4
FSD2		1,27	0,36	0,07	4

Fig. S5B

added prot	amount (µg)	average	sd	p-value	n
GST	0	1	0		3
	0,5	0,92	0,18	0,33	3
	1	0,97	0,03	0,54	3
	2	1,10	0,23	0,41	3
	3	0,91	0,15	0,07	3
MBP-NIMIN1	0	1	0		3
	0,5	0,81	0,25	0,42	3
	1	0,90	0,17	0,62	3
	2	0,96	0,11	0,69	3
	3	0,93	0,26	0,11	3
GST-LSU2	0	1	0		3
	0,5	1,07	0,18	0,28	3
	1	1,24	0,19	0,05	3
	2	1,25	0,12	0,01	3
	3	1,62	0,19	0,00	3

Fig. S6

genotype	Time-point	average	sd	p-value	n
WT	0 hpi +S	0,42	0,03		3
amiR-LSUa	0 hpi +S	0,44	0,03		3
amiR-LSUb	0 hpi +S	0,46	0,04		3
amiR-LSUc	0 hpi +S	0,43	0,03		3

WT	1 hpi +S	0,23	0,02		3
amiR-LSUa	1 hpi +S	0,24	0,00	0,15	3
amiR-LSUb	1 hpi +S	0,24	0,03	0,33	3
amiR-LSUc	1 hpi +S	0,25	0,06	0,26	3
WT	4 hpi +S	0,40	0,04		3
amiR-LSUa	4 hpi +S	0,38	0,05	0,34	3
amiR-LSUb	4 hpi +S	0,33	0,01	0,12	3
amiR-LSUc	4 hpi +S	0,43	0,06	0,24	3
WT	0 hpi -S	0,21	0,03		3
amiR-LSUa	0 hpi -S	0,37	0,05	0,00	3
amiR-LSUb	0 hpi -S	0,37	0,03	0,00	3
amiR-LSUc	0 hpi -S	0,37	0,01	0,00	3
WT	1 hpi -S	0,26	0,04		3
amiR-LSUa	1 hpi -S	0,28	0,01	0,25	3
amiR-LSUb	1 hpi -S	0,25	0,01	0,43	3
amiR-LSUc	1 hpi -S	0,23	0,01	0,12	3
WT	4 hpi -S	0,23	0,02		3
amiR-LSUa	4 hpi -S	0,27	0,01	0,01	3
amiR-LSUb	4 hpi -S	0,26	0,03	0,06	3
amiR-LSUc	4 hpi -S	0,29	0,02	0,01	3

Fig. S7

genotype	condition	average	sd	p-value	n
WT	DC3000 plus S	7,29	0,13		4
fsd2-2	DC3000 plus S	7,35	0,23	0,31	4
WT	DC3000 minus S	7,61	0,11		4
fsd2-2	DC3000 minus S	7,76	0,09	0,05	4

Table S2. Oligonucleotides used in the present work. The name, sequence (5'-3') and target for each oligonucleotide are provided.

**name**	**Sequence (5'-3')**	**Target**
amiRNA-A	GGGGACAAGTTTGTACAAAAAAGCAGGCTT CCTGCAAGGCGATTAAGTTGGGTAAC	Cloning amiRNA
amiRNA-B	GGGGACCACTTTGTACAAGAAAGCTGGGTC TCAGCGGATAACAATTTCACACAGGAAACA G	Cloning amiRNA
amiRNA-LSU1,3-Ia	GATAAAGACTCGACCCCTAGCTTTCTCTCTT TTGTATTCC	amiR-LSUa
amiRNA-LSU1,3-IIa	GAAAGCTAGGGGTCGAGTCTTTATCAAAGA GAATCAATGA	amiR-LSUa
amiRNA-LSU1,3-IIIa	GAAAACTAGGGGTCGTGTCTTTTTCACAGG TCGTGATATG	amiR-LSUa
amiRNA-LSU1,3-Iva	GAAAAAGACACGACCCCTAGTTTTCTACAT ATATATTCCT	amiR-LSUa
amiRNA-LSU1,3-Ib	GATAAAGACTCGACCTCCCGCGCTCTCTCTT TTGTATTCC	amiR-LSUb
amiRNA-LSU1,3-IIb	GAGCGCGGGAGGTCGAGTCTTTATCAAAGA GAATCAATGA	amiR-LSUb
amiRNA-LSU1,3-IIIb	GAGCACGGGAGGTCGTGTCTTTTTCACAGG TCGTGATATG	amiR-LSUb
amiRNA-LSU1,3-IVb	GAAAAAGACACGACCTCCCGTGCTCTACAT ATATATTCCT	amiR-LSUb
amiRNA-LSU2,4-Ib	GATAGAGTGGTAATCACGTGCTTTCTCTCTT TTGTATTCC	amiR-LSUc
amiRNA-LSU2,4-Iib	GAAAGCACGTGATTACCACTCTATCAAAGA GAATCAATGA	amiR-LSUc
amiRNA-LSU2,4-IIIb	GAAAACACGTGATTAGCACTCTTTCACAGG TCGTGATATG	amiR-LSUc
amiRNA-LSU2,4-Ivb	GAAAGAGTGCTAATCACGTGTTTTCTACAT ATATATTCCT	amiR-LSUc
LSU1-qPCR-F	GAGGCGGAAGAGCAACTCTG	qPCR LSU1
LSU1-qPCR-R	CCATGAGGAAGAGCATGCGA	qPCR LSU1
LSU2-qPCR-F	GGCTCGTGATTACCACTCTCG	qPCR LSU2
LSU2-qPCR-R	CTACGGAGAGGCAGAGGCAG	qPCR LSU2
LSU3-qPCR-F	CAAGAAGCAGCAAAACCACCT	qPCR LSU3
LSU3-qPCR-R	CCACCGTCACATAACCTCCT	qPCR LSU3
LSU4-qPCR-F	TCGTGATTACCACACTCGCAT	qPCR LSU4
LSU4-qPCR-R	TTCTAGGGAGAGGCAGAGTCG	qPCR LSU4
UBQ10-qPCR-F	GGCCTTGTATAATCCCTGATGAATAAG	qPCR UBQ10
UBQ10-qPCR-R	AAAGAGATAACAGGAACGGAAACATAGT	qPCR UBQ10
ACT2-qPCR-F	CTTGCACCAAGCAGCATGAA	qPCR ACT2
ACT2-qPCR-R	CCGATCCAGACACTGTACTTCCTT	qPCR ACT2
ACT8-qPCR-F	AGCTCCGTATTGCTCCTGAA	qPCR ACT8
ACT8-qPCR-R	CAGTAAGGTCACGACCAGCA	qPCR ACT8
EF1-qPCR-F	TGGTGACGCTGGTATGGTTA	qPCR EF1
EF1-qPCR-R	TCCTTCTTGTCCACGCTCTT	qPCR EF1

Table S3. Primary antibodies used in the present work. The name, dilution and secondary antibody are provided.

Name	Dilution	Secondary
α -MBP (New England)	1:10.000	α -IgG mouse
α -UGPase (Agrisera)	1:1000	α -IgG rabbit
α -V-ATPase (Agrisera)	1:2.500	α -IgG rabbit
α -H3 (Abcam)	1:10.000	α -IgG rabbit
α -HA-HRP (Roche)	1:1.500	-
α -GFP (Life Technologies)	1:3.000	α -IgG mouse
